# Multi-omics analysis reveals the mechanism underlying microbial-driven flavor formation in fermented xuecai (*Brassica juncea* var. *multiceps*)

**DOI:** 10.3389/fmicb.2026.1784956

**Published:** 2026-03-23

**Authors:** Mengqin Cao, Haibo Hao, Shuya Duan, Zitong Meng, Ruixin Li, Jiwei Jin, Yong Luo, Xiliang Ren, Bo Sun, Yishan Song, Dingyu Zhang, Hongfang Zhu

**Affiliations:** 1 College of Food Science and Technology, Shanghai Ocean University, Shanghai, China; 2 Shanghai Key Laboratory of Protected Horticultural Technology, Horticultural Research Institute Shanghai Academy of Agricultural Sciences, Shanghai, China; 3 Institute of Edible Fungi, Shanghai Academy of Agricultural Sciences, Shanghai, China; 4 Ningbo Academy of Agricultural Sciences, Ningbo, China; 5 College of Horticulture, Sichuan Agricultural University, Chengdu, China

**Keywords:** co-occurrence network, fermented xuecai, flavor substance, free amino acids, metabolomics, microbiomics

## Abstract

The unique flavor of xuecai stems from microbial metabolism, but how microbial community evolution is connected to fermentative flavor formation remains unclear. In this study, a comprehensive analysis of xuecai fermentation was conducted via untargeted metabolomics, microbiomics, and biochemical techniques to investigate the relationships among its physicochemical properties, flavor compounds, and microbial communities. Nontargeted metabolomics revealed 113 volatile flavor compounds, including 14 aldehydes, 38 alcohols, 37 esters, 11 ketones, and 13 phenols, and the overall flavor and nutritional quality of xuecai reached an optimal state after 32 days of fermentation. A correlation analysis of the microbial communities and physicochemical indicators showed that *Companilactobacillus*, *Weissella*, *Latilactobacillus*, *Debaryomyces*, and *Hannaella* were the core microbial taxa driving flavor formation; specifically, *Companilactobacillus*, *Weissella*, and *Debaryomyces* exhibited positive correlations with 3 volatile flavor compounds and 15 amino acids. Furthermore, structural equation modeling (SEM) demonstrated the direct role of microbial communities in shaping the flavor profile of fermented xuecai. This study reveals the dynamic mechanism underlying flavor formation during xuecai fermentation and provides new insights into microbe-mediated fermentation process optimization in industrial production.

## 1 Introduction

Fermentation is the process of preparing specific food products through the metabolic activity of microorganisms, which not only enhances the flavor profile of these food products but also increases their nutritional value (Xing et al., 2025; Zhang et al., 2025). A wide variety of foods have been fermented, including fruits and vegetables, grains, meat, fish, and dairy products. However, fermented vegetables are widely consumed because of their low cost, unique flavor and high nutritional value (Bernacka et al., 2025; Knez et al., 2023; Yuan et al., 2023). Xuecai (*Brassica juncea* var. *multiceps*) is a primary processing vegetable cultivated extensively throughout China’s Yangtze River Basin and is characterized by an annual production of 1–2 million tons and a pickled product market valued at 18 billion RMB in 2019. Fermented xuecai is rich in vitamin C, flavonoids, glucosinolates, fatty acids, and flavor compounds, notably amino acids and sulfides, which are favored by consumers (Harbaum et al., 2008; Ibrahim et al., 2023; Martinez-Villaluenga et al., 2009).

The pickling process of fermented vegetables involves complex biochemical reactions. Microbial metabolic activity is a key factor driving quality evolution and the formation of characteristic flavor compounds in fermented vegetables (Major et al., 2022; Zhou et al., 2024). The brine of aged fermented vegetables is abundant in microorganisms, such as beneficial lactic acid bacteria (LAB) and yeasts (Liu et al., 2021). Lactic acid bacteria (LAB) produce large volumes of lactic acid by metabolizing sugars in vegetables, thereby lowering the pH value of the environment, which can effectively suppress the proliferation of spoilage bacteria and pathogenic species. Carbapenem-resistant gram-negative bacteria (CR-GNB) are a group of bacteria that pose a significant threat to human health, and drug therapy can be used to treat infections caused by these bacteria (Zhuang et al., 2023). In addition to drug treatment, microorganisms can also exert inhibitory effects on pathogenic bacteria. For example, some beneficial lactic acid bacteria, such as LAB, can effectively inhibit the growth and adhesion of oral pathogenic bacteria, thereby improving human oral health (Squarzanti et al., 2024). In addition, several beneficial gut microbes can drive the regulation of intestinal barrier function and the production of bioactive metabolites. *Lactobacillus* and *Bifidobacterium* strains directly regulate barrier function by upregulating the mRNA expression of tight junction proteins (Bock et al., 2024). Astragali Radix can modulate the gut microbiota, enhance the immune barrier function of the intestinal mucosa, and control diabetes by increasing tyrosine kinase activity (Su et al., 2023).

During vegetable fermentation, yeasts not only increase the levels of umami and sweet amino acids but also produce aromatic compounds such as phenethyl alcohol and linalool, which play key roles in the development of the characteristic aroma of fermented vegetables (Wang R. et al., 2024). Moreover, the formation of flavor substances in fermented vegetables is significantly influenced by the nutrients within the vegetable tissue. This characteristic is exemplified by mustard greens (*Brassica juncea*), which contain high levels of glucosinolates. During fermentation, microorganisms decompose these glucosinolates into sulfur-containing compounds, including isothiocyanates and thiocyanates, thereby ameliorating the pungent flavor of pickled vegetables produced by fermentation (Fang et al., 2008). Microorganisms can also use sulfur-containing amino acids in vegetables for catabolism to produce sulfur compounds such as hydrogen sulfide, which gives pickles their distinctive aroma (Landaud et al., 2008). Recent studies have reported correlations between flavor compounds and key microorganisms during the fermentation of vegetables such as radish, cabbage and chili pepper (Sun et al., 2023; Xu et al., 2021; Yang et al., 2022). However, most of these investigations have been limited to bacterial associations. Owing to the complex nutritional composition of xuecai, the relationships between flavor formation and both fungal and bacterial communities during xuecai fermentation remain unclear.

With the development and application of multi-omics technologies, high-throughput sequencing has enabled the identification of the diversity and composition of microorganisms in fermented vegetable brine. A typical example is found in fermented Chinese artichoke, where *Lactiplantibacillus*, *Pediococcus*, and *Bacillus* are the key genera contributing to flavor development (Peng et al., 2025a). An analysis of the microbiota of fermented broad beans revealed 66 key microorganisms, of which *Lactobacillus*, *Weissella* and *Pichia* were identified as the core microorganisms driving fermentation (Liu et al., 2020). Furthermore, metabolomics is key for identifying the composition of flavor compounds in fermented vegetables and has been widely applied in the study of fermented chayote (Shang et al., 2022), Ma bamboo shoots (Hu et al., 2023), and chili pepper.

In this study, we analyzed the structure and composition of the fermentation microbiota via amplicon sequencing. Biochemical analyses were conducted to detect changes in pH, salt concentration, and the activities of key enzymes throughout the fermentation process. Additionally, we investigated the changes in metabolites and flavor components via gas chromatography–time-of-flight–mass spectrometry (GC–TOF–MS), along with analyses of amino acids and organic acids. Finally, the correlation analysis revealed the dominant microbial species that were significantly associated with flavor formation. These results are expected to contribute to improving quality in standardized production and may serve as a reference for the research and development of microbial communities for the fermentation of other vegetables.

## 2 Materials and methods

### 2.1 Xuecai fermentation and sample collection

The xuecai used in this study was collected from Lao Gang town, Shanghai (southeastern Pudong New Area, Shanghai, China; 31.03° N, 121.78° E). Fresh, pest-free, and uniformly sized xuecai was air-dried for one day and then placed in a fermentation tank. Salt was added at a ratio of 10:1 (cabbage/salt), with alternating layers of xuecai and salt. The mixture was pressed with a heavy weight for 24 h until the moisture was released. The dehydrated xuecai was then transferred to a clean and dry fermentation tank for sealed fermentation (Zhao et al., 2007). In our study, traditional spontaneous fermentation was applied without any starter cultures. As a result, fermented xuecai requires 1–2 months from the initial stage to full maturity. Since metabolites change rapidly during the early fermentation stage, short sampling intervals were adopted. In contrast, longer intervals were used in the late stage, as the variations in most components gradually slowed and stabilized. Therefore, we collected the samples at different time points during fermentation (Days 4, 8, 16, 32, and 64). At each time point, the xuecai and fermentation broth were collected from the top, middle, and lowest parts of the fermentation tanks, thoroughly mixed to form a composite sample, and stored at −80 °C. Six biological replicates were prepared at every time point. The xuecai samples were subsequently used for the physicochemical analysis and metabolomic analysis, while the fermentation broth was used for the microbial community analysis.

### 2.2 Determination of physicochemical indicators

Titratable acidity (TA) in the samples was determined by titration with 0.01 mol/L NaOH to a pH of 8.2 ± 0.2 (Yang et al., 2022). The pH of the samples was determined with a pH meter (Huang et al., 2022). The nitrite content was determined in accordance with the national standards of China (GB 5009.33–2016). The activities of nitrite reductase, glutamate synthetase and lactate dehydrogenase in xuecai were determined using kits provided by Suzhou Keming Biotechnology Co., Ltd. (Suzhou, China), and triplicate measurements were performed for each sample.

### 2.3 Sensory analysis of fermented xuecai

The electronic tongue system was used to assess the taste characteristics of xuecai at different fermentation stages. The electronic tongue was equipped with nine sensors: bitterness, sourness, saltiness, umami, sweetness, astringency, richness, aftertaste-A and aftertaste-B (Liu et al., 2024). Electronic nose detection was conducted via direct headspace sampling (Chen et al., 2023; Luo et al., 2025). Each sample was analyzed in five replicates.

### 2.4 Analysis of amino acids and organic acids

The determination of amino acids and organic acids was conducted following previous methods (Yang et al., 2022) with modifications. The contents of the substances were quantified by ultra-performance liquid chromatography–tandem mass spectrometry (UPLC–MS/MS). Standard amino acids and organic acids were obtained from Yuanye Biotechnology Co., Ltd. (Shanghai, China).

### 2.5 Untargeted metabolomic analysis relying on liquid chromatography–mass spectrometry

We weighed 38–43 mg of ground sample in a 1.5 mL centrifuge tube, after which 300 μL of precooled methanol (containing 5 ppm 2-chlorophenylalanine) was added, followed by vortexing for 30 s. After sonication for 10 min, the sample was frozen at −20 °C for 30 min. Afterwards, centrifugation was performed at 12,000 rpm and 4 °C for 10 min. The supernatant obtained was passed through a 0.22 μm membrane filter for filtration for subsequent determination (Vasilev et al., 2016). An aliquot (10–20 μL) of each filtrate was collected and pooled to prepare a composite quality control (QC) sample. Analyses of test samples and QC samples were performed using a Thermo Vanquish UHPLC system equipped with an ACQUITY UPLC HSS T3 column. Prior to the analytical runs, the system was conditioned with three consecutive injections of the QC samples to ensure stability. During the batch analysis, one QC sample was inserted after every 5 test samples to monitor instrumental drift. Intrabatch correction was applied to the QC data using the SERRF algorithm. Metabolomic profiling of the fermentation samples was performed by Personalbio Biotechnology Co., Ltd. (Shanghai, China). Metabolites were annotated by searching the Personalbio Next-Generation Metabolomics (PSNGM) database. The acquired mass spectrometry data were matched with both in-house standard libraries and public databases using MS1 and MS2 spectra. All the annotated metabolites were assigned a confidence level of 2 or higher. Key parameters for the database search were set as follows: MS1 mass tolerance of 0.01 Da; MS2 mass tolerance of 0.05 Da; smoothing level of 3; minimum peak height of 10,000; minimum peak width of 5; mass slice width of 0.05 Da; and identification score threshold of 70.

### 2.6 Untargeted metabolomic analysis relying on gas chromatography–time–of–flight–mass spectrometry

The analysis of samples was performed using gas chromatography–mass spectrometry (GC–MS) with a Shimadzu system. The method was modified from a previous report (Peng et al., 2025a): 50 mg of sample and 500 μL of extraction solution were added to a 1.5 mL centrifuge tube. The mixture was ground with a tissue grinder for 5 min and then sonicated in ice water for 5 min; this process was repeated three times. The sample was subsequently centrifuged at 12,000 rpm and 4 °C for 13 min. A 200 μL aliquot of the supernatant was transferred to a fresh centrifuge tube, and 50 μL of the supernatant from each sample was pooled to form a QC sample. Finally, 60 μL of BSTFA and 5 μL of FAMEs were added to the dried individual samples and the pooled sample, respectively. All the samples were analyzed using a GC–MS system fitted with a DB-5MS capillary column (Dunn et al., 2011; Kind et al., 2009). The samples were analyzed using the LECO-FiehnRtx5 database. Metabolites were identified based on similarity scores, where higher values indicate better matches. The mass scan range was 20–400, the signal-to-noise ratio was set to 10, and the peak width was 3–4 s.

### 2.7 Microbiome analysis

We extracted total DNA from xuecai at different fermentation time points using the MagBeads FastDNA Kit for Soil (116564384) (MP Biomedicals, CA, United States) and determined the molecular size and quantity of the isolated DNA via 0.8% agarose gel electrophoresis and Nanodrop. Bacterial PCR amplification and sequencing were performed using 16S rRNA V3-V4 region-specific primers. The fungal ITS region was amplified and sequenced using the ITS5 (5′-GGAAGTAAAAGTCGTAACAAGG-3′) and ITS2 (5′-GCTGCGTTCTTCATCGATGC-3′) primers. Library was constructed with the Illumina TruSeq Nano DNA LT Library Preparation Kit. Library quantification was performed using a Qubit 4, and the fragment size distribution of the PCR-enriched library was analyzed on an Agilent 2100 Bioanalyzer for quality control, and the final library pool (10 nM) was attenuated to an appropriate concentration for subsequent sequencing. Subsequently, the Illumina NovaSeq instrument was used for 2 × 250 bp paired-end sequencing with the NovaSeq 6000 SP Reagent Kit. Analysis of Microbial community composition was performed using QIIME2 version 2024.5 (Bolyen et al., 2019). The raw reads were subjected to primer trimming and demultiplexing using the Cutadapt and Demux plugins, respectively, after which the sequences are processed using the DADA2 plugin. Through the use of the Greengenes database, ASV feature sequences were matched against the reference sequences in the database, enabling the acquisition of taxonomic information for each ASV. The abundance matrix from which rare ASVs had been removed was then used for a succession of subsequent analyses (Bokulich et al., 2018; Kõljalg et al., 2013). Alpha diversity (Chao, 1984; Simpson, 1949), beta diversity (Bray and Curtis, 1957; Jaccard, 1908; Lozupone and Knight, 2005), and between-group differences analyses were performed via QIIME2 software and R software.

### 2.8 Statistical analysis

Three experimental replicates were conducted for the samples at each fermentation time point. The values of total acidity, pH, salinity, nitrite, enzyme activity and nonvolatile compounds are shown as the means ± standard deviations. The data were plotted using GraphPad Prism (version 10). Statistical comparisons between the two groups were performed with IBM SPSS (version 27.0) using one-way analysis of variance followed by Duncan’s *post hoc* test (*p* < 0.05). Metabolite and the microbial community composition analyses were performed on the Personalbio Cloud platform.^1^ Analyses were performed using full linear discriminant analysis (LDA) effect size (LEfSe) to determine potential microbial markers. The relationships between the microbial communities and physicochemical indicators were investigated by performing a redundancy analysis (RDA) and the Mantel test. The correlations between distinct flavor compounds and dominant bacteria were examined using Spearman’s correlation analysis. A network diagram of microbial associations with flavor compounds was constructed with the Gephi tool (Gephi–The Open Graph *Viz* Platform). Partial least squares path modeling (PLS–PM) SEM structural equation analyses were performed with the plspm package (0.5.1) in R (4.3.3) software. Model fit was judged according to the following criteria: a nonsignificant chi-square test (*p* > 0.05) and a high goodness-of-fit index (GOF > 0.60) (Hair et al., 2011; Hao et al., 2024).

## 3 Results

### 3.1 Analysis of physicochemical indicators during the fermentation of xuecai

**Figure 1:**
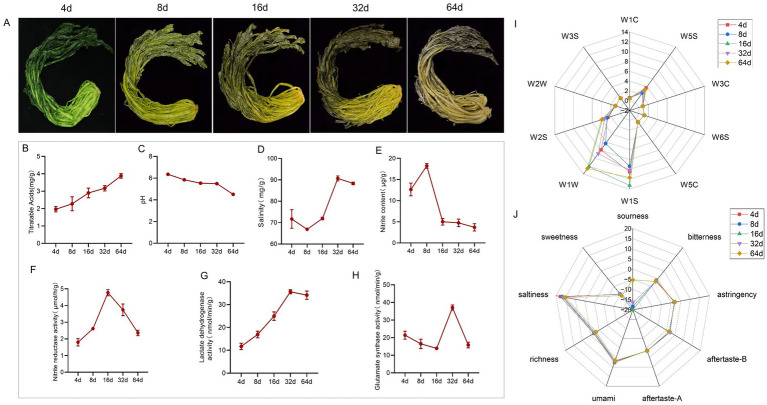
Changes in physiochemical indicators and the sensory analysis of *xuecai* during the fermentation process. **(A)** Appearance of xuecai at different fermentation time points. **(B)** Changes in the levels of titratable acidity, **(C)** pH, **(D)** salinity, **(E)** nitrite, **(F)** nitrite reductase activity, **(G)** lactate dehydrogenase activity, and **(H)** glutamate synthase activity. **(I)** Radar map of the results obtained from the electronic nose (W1C, aromatic compounds; W5S, nitrogen oxide; W3C, ammonia and aromatic compounds; W6S, hydride; W5C, alkanes and aromatic compounds; W1S, methane; W1W, sulfides and terpenes; W2S, alcohols, aldehydes, and ketones; W2W, aromatic components and organic sulfides; W3S, long-chain alkanes). **(J)** Results obtained from the electronic tongue.

Flavor profiles formed during vegetable fermentation are determined mainly by the microbial communities involved in the fermentation process, whose composition is susceptible to regulation by a variety of environmental parameters, including key factors such as acidity (pH), the titratable acidity (TA) and salt concentration (Wang et al., 2023). The results showed that fresh xuecai gradually took on a yellow to yellow-brown color, and the TA level gradually increased, reaching 3.89 mg/g by 64 d (Figures 1A,B). Moreover, the pH also gradually decreased (Figure 1C). The results indicated that the overall salinity of the fermented xuecai gradually increased (Figure 1D), whereas the nitrite content tended to increase initially but then decrease (Figure 1E). The activity of nitrite reductase (NiR) initially increased but then decreased (Figure 1F), and the synthesis of nitrite from 4 d to 16 d of fermentation induced the production of NiR in microbes such as LAB; in the later stages, the enzyme activity gradually decreased because of reduced nitrite availability. The LDH activity of xuecai continued to increase during fermentation, peaking at 32 d and then decreasing (Figure 1G), demonstrating the important role of LAB in the fermentation process. Additionally, the enzyme activity of glutamate synthase increased sharply from 16 d to 32 d, after which it decreased (Figure 1H).

Xuecai appears to have a variety of flavor characteristics at different fermentation stages. Electronic noses can detect the odor profiles of flavor compounds in food products and present them as data (Chen et al., 2023). The flavor substances detected by each sensor are shown in Supplementary Table S1. The flavors of xuecai at different fermentation time points are shown, with the W2S, W1S and W1W sensors showing significant differences and the remaining sensors showing almost no change. The response value of the W1W sensor increased from 32 d to 64 d, whereas that of the W2S sensor was the greatest at 32 d and 64 d (Figure 1I). This pattern indicates the accumulation of sulfur compounds, alcohols, aldehydes, and ketones in the later stages of fermentation, which contributes to flavor development. The electronic tongue analysis revealed that all flavor attributes except sourness exhibited virtually no change throughout the fermentation process (Figure 1J); in particular, sourness increased continuously.

### 3.2 Analysis of nonvolatile compounds during xuecai fermentation

**Figure 2:**
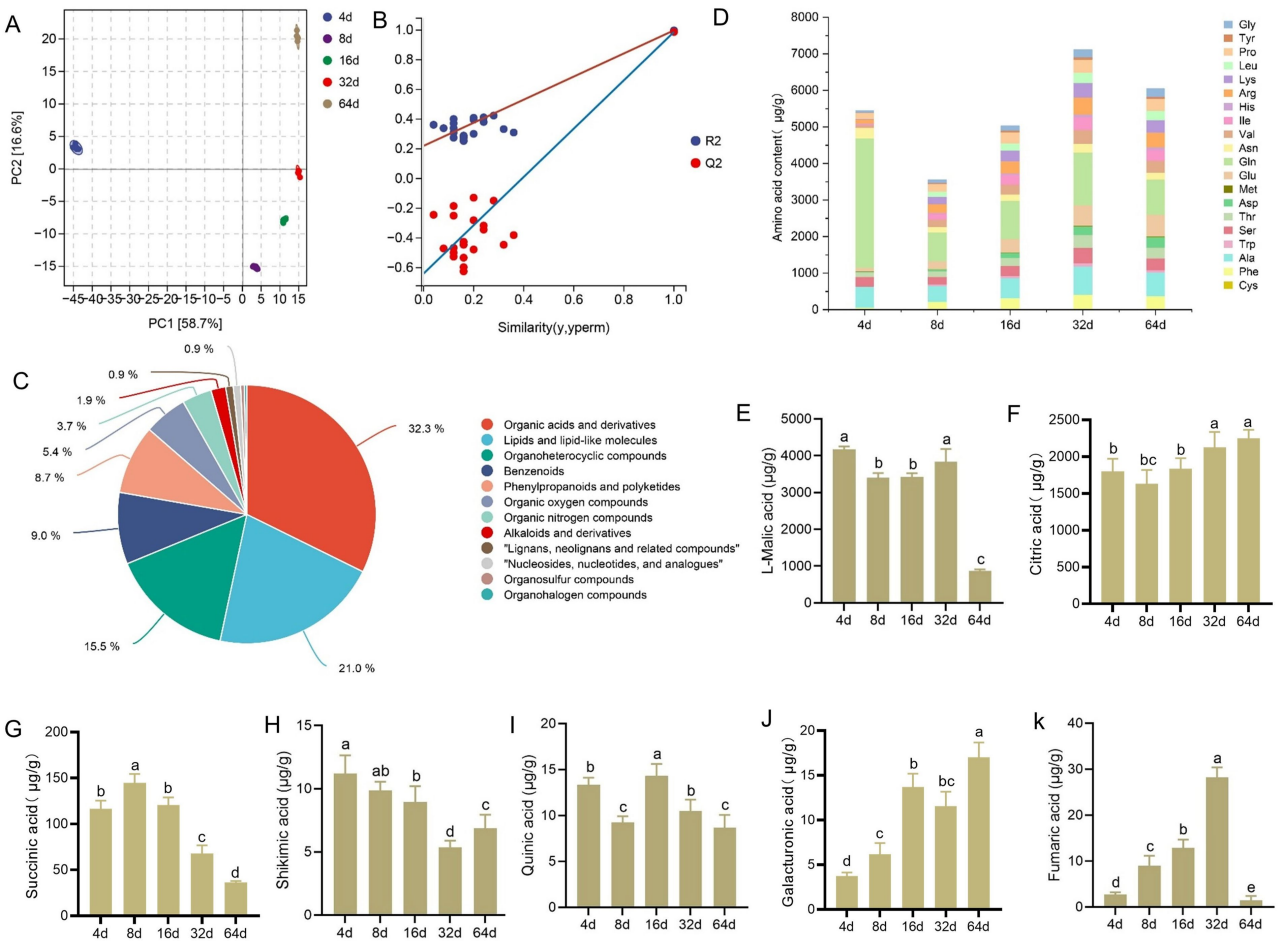
Changes in the levels of nonvolatile compounds during xuecai fermentation. **(A)** Principal component analysis (PCA) of nonvolatile compounds. **(B)** Plot showing the validation of the PLS-DA model. **(C)** Classification of all detected nonvolatile metabolites in fermented xuecai samples. **(D)** Changes in the levels of amino acids, **(E)** L-malic acid, **(F)** citric acid, **(G)** succinic acid, **(H)** shikimic acid, **(I)** quinic acid, **(J)** galacturonic acid, and **(K)** fumaric acid. Statistical significance is denoted by different letters (*p* < 0.05).

The multivariate statistical analysis showed that the first two principal components explained 75.3% of the total variance, with the five sample groups distributed across three distinct quadrants. This result indicated significant variability in the metabolite composition among samples at different fermentation time points (Figure 2A). Partial least squares discriminant analysis (PLS–DA) was used to perform a permutation test for the overall sample, and the results showed that the PLS-DA model exhibited strong fitting performance and predictive capability, and the rightmost original Q2 point was higher than all the other Q2 points (Figure 2B). A total of 647 compounds, including organic acids and their derivatives (209); lipids and lipid-like molecules (136); organic heterocyclic compounds (100); benzenoids (58); phenylacetones and polyketides (56); organic oxygen compounds (35); organonitrogen compounds (24); alkaloids and their derivatives (12); lignans, neolignans and their related compounds (6); nucleosides, nucleotides, and their analogs (6); organosulfur compounds (3) and organic halogen compounds (2) (Figure 2C), were detected on 4, 8, 16, 32, and 64 d of fermentation. To better understand the changes in metabolites in xuecai at different fermentation time periods, we conducted the hierarchical clustering analysis (HCA) based on the experimental results, and accordingly generated heatmap (Supplementary Figure S1) revealed that the metabolite aggregation of the samples at 4 d differed significantly from that at the other four time periods, whereas the metabolite aggregation of the samples at 8, 16, and 32 d was similar, and the trend was consistent with the results obtained from PCA.

During vegetable fermentation, free amino acids (FAAs) and organic acids not only regulate the metabolic activities of microorganisms and provide nutrients but also serve as key flavor compounds (Ye et al., 2022). Amino acids are categorized as umami, sweet, or bitter based on their flavor characteristics. As shown in Figure 2D, the levels of most of the amino acids tended to increase overall during fermentation, and the concentrations of umami amino acids such as L-aspartic acid and L-glutamic acid continued to increase, with the concentration of L-aspartic acid increasing from the initial level of 12.18 μg/g to 284.29 μg/g at the later stage and that of L-glutamic acid increasing from 94.11 μg/g at 4 d to 588.84 μg/g at 32 d. Sweet amino acids such as L-alanine, L-serine, L-threonine and L-proline were the most abundant at 32 d, and the concentrations of bitter amino acids such as L-leucine, L-valine and L-phenylalanine also tended to increase overall, with the highest levels occurring at 32 d and gradually decreasing thereafter at 64 d.

We detected several organic acids in the fermentation products; L-malic acid and citric acid were the major organic acids measured during the fermentation process, with concentrations of 4,175 μg/g and 1,800 μg/g at the initial stage of fermentation, respectively (Figures 2E,F). In addition, the contents of succinic acid and shikimic acid decreased gradually (Figures 2G,H). The content of quinic acid reached the maximum value at 16 d and then gradually decreased (Figure 2I). In contrast, the galacturonic acid content increased progressively (Figure 2J), and the fumaric acid content also increased, reaching its maximum at 32 d, followed by a rapid decrease (Figure 2K).

### 3.3 Analysis of volatile flavor compounds during xuecai fermentation

**Figure 3:**
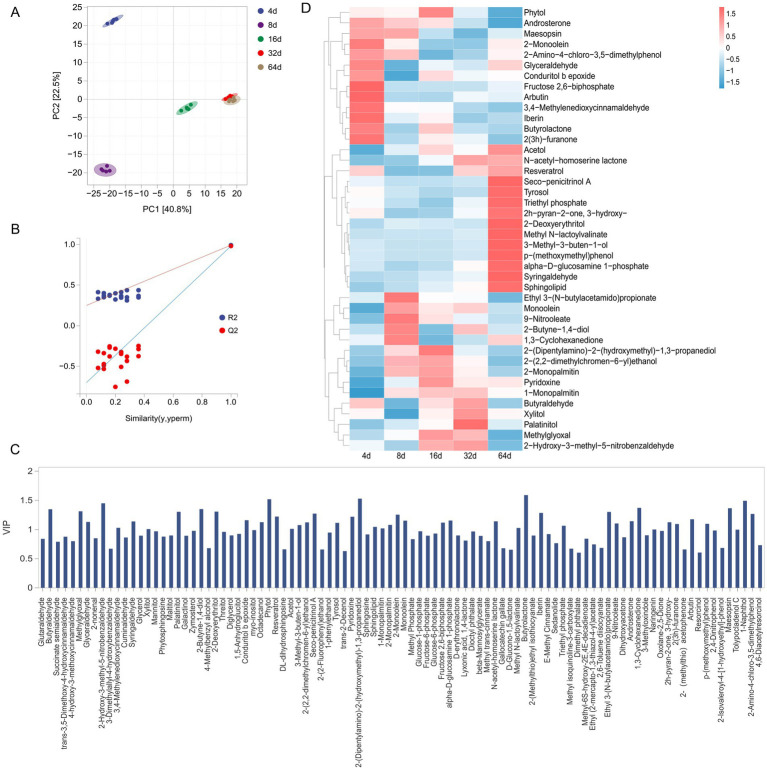
Changes in the levels of volatile compounds during the fermentation of xuecai. **(A)** Principal component analysis (PCA) of volatile compounds during fermentation. **(B)** Plot showing the validation of the PLS-DA model. **(C)** VIP scores of the VOCs (*p* < 0.05). **(D)** Changes in the differential VOC content during fermentation.

GC–TOF–MS was used to analyse the changes in metabolic substances during xuecai fermentation. PCA indicated that PC1 and PC2 accounted for 22.5 and 40.8% of the variance, respectively. Except for the samples at 32 d and 64 d, which were not significantly different, the remaining samples were relatively distant from one another (Figure 3A), possibly because the metabolic activities of the microorganisms tended to equilibrate at the latter stage of fermentation. Thus, no notable changes in metabolites were observed. The replacement test plot for the overall samples also revealed that the PLS–DA model exhibited strong fitting performance and predictive capability (Figure 3B).

We detected 113 volatile compounds, including 14 aldehydes, 38 alcohols, 37 esters, 11 ketones and 13 phenols, during the fermentation of xuecai. A VIP > 1 is often used as a criterion for screening significant flavor compounds (Giannetti et al., 2017). We obtained 46 different metabolites, such as N-acetyl-homoserine lactone, phytol, syringaldehyde, 3,4-methylenedioxycinnamaldehyde, butyrolactone, and 2(3H)-furanone (VIP > 1, *p* < 0.05) (Figure 3C). Among these metabolites, the contents of glyceraldehyde, fructose 2,6-biphosphate, 3,4-methylenedioxycinnamaldehyde, iberin, butyrolactone, 2(3H)-furanone, and other compounds gradually decreased during fermentation (Figure 3D; Supplementary Table S2). The contents of alcohols and esters, such as pyridoxine, acetol, tyrosol, N-acetyl-homoserine lactone (AHL), 2H-pyran-2-one-3-hydroxy, 2-deoxyerythritol, methyl N-lactoylvalinate, 3-methyl-3-buten-1-ol, syringaldehyde, resveratrol, and other flavor compounds, gradually increased during the later stages of fermentation (Figure 3D). 2H-Pyran-2-one-3-hydroxy is an organic compound of the pyranone class that first decreased but then increased during fermentation, peaking at 64 d.

### 3.4 Metabolic analysis of amino acid and organic acid synthesis during xuecai fermentation

**Figure 4:**
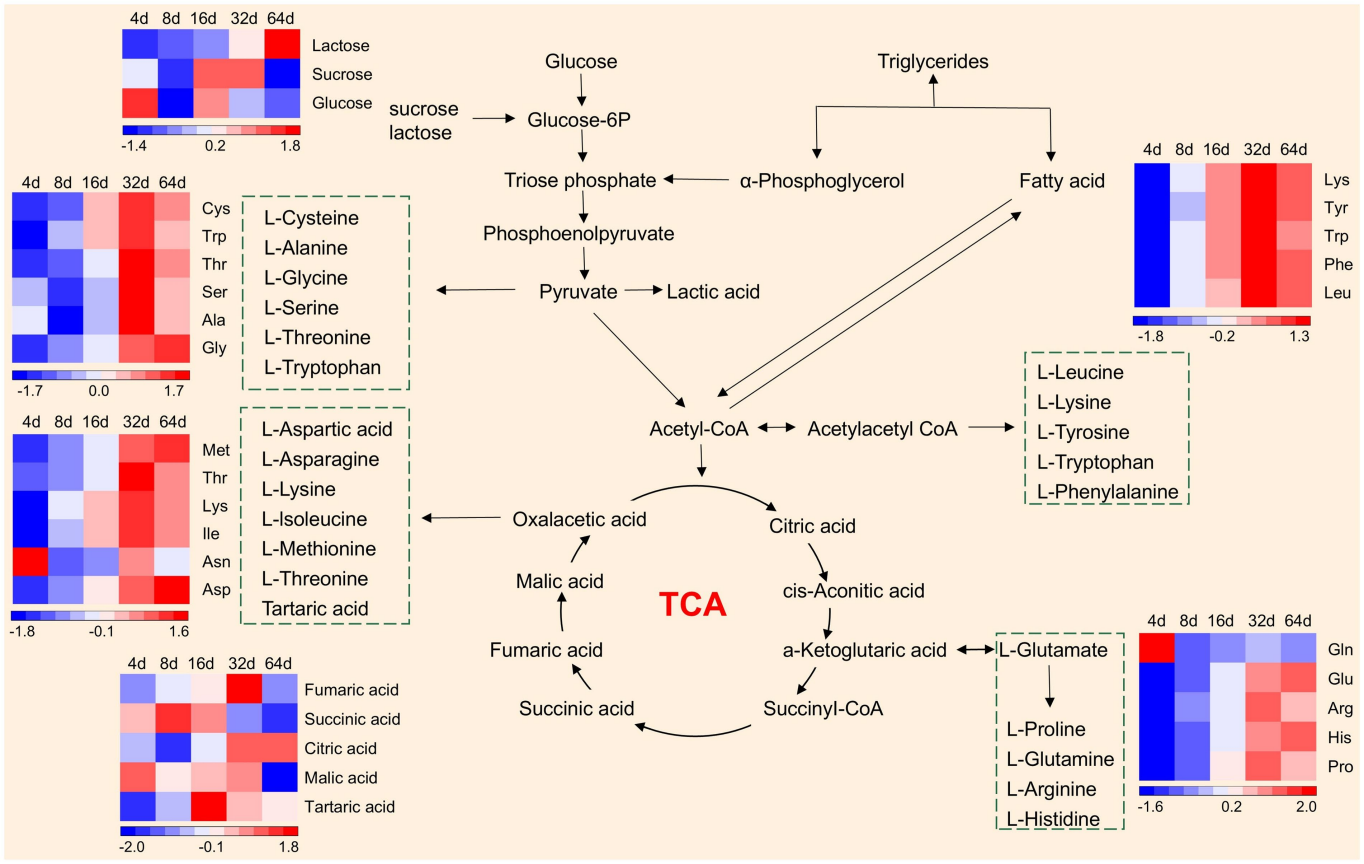
Analysis of amino acid and organic acid metabolic pathway during xuecai fermentation.

Sugars are the basic substances for the survival of fermenting microorganisms and the transformation of flavor components (Athanasiadis et al., 2001). The lactose content gradually increased during fermentation, and sucrose and glucose were higher at 4 d but were then gradually consumed by microorganisms to a lower level after 16 d (Figure 4). Glucose is converted into glucose 6-phosphate and triose phosphate, which are further metabolized into pyruvate (Lee et al., 1973). Pyruvate partly produces lactic acid and partly participates in the TCA cycle in the presence of acetyl-CoA to produce organic acids. Lactic acid is a common organic acid produced during vegetable fermentation; in addition, other organic acids, such as fumaric acid, malic acid, and citric acid, are present. The contents of malic acid, succinic acid and fumaric acid gradually decreased during the later stages of fermentation. These organic acid intermediates were eventually converted to amino acids such that their contents decreased gradually in the latter stages of fermentation. The results also revealed that the contents of the various flavor-related amino acids continued to increase and peaked at 32 d but decreased at 64 d.

During food fermentation, under the action of proteinases secreted by LAB, proteins can be hydrolysed into peptides and free amino acids (de Viana Souza and Silva Dias, 2017). Free amino acids act as flavor precursors, generating sulfur-containing compounds via elimination reactions and thiols via carbon-sulfur lyases. Our results revealed that the contents of thiols and sulfides gradually increased during fermentation (Supplementary Figure S2), providing a unique flavor to fermented xuecai. In addition, the aminotransferase produced by LAB converts α-ketoglutarate to glutamate and amino acids to α-keto acids. Generated α-keto acids formed aldehydes via decarboxylase, aldehydes yielded carboxylic acids and alcohols (via dehydrogenase), and yeast serves as the primary source of esterase, which catalyzes the esterification reaction between alcohols and carboxylic acids to produce esters (Supplementary Figure S2). Both esters and alcohols are important flavor components of fermented xuecai.

### 3.5 Changes in microbial community dynamics during xuecai fermentation

**Figure 5:**
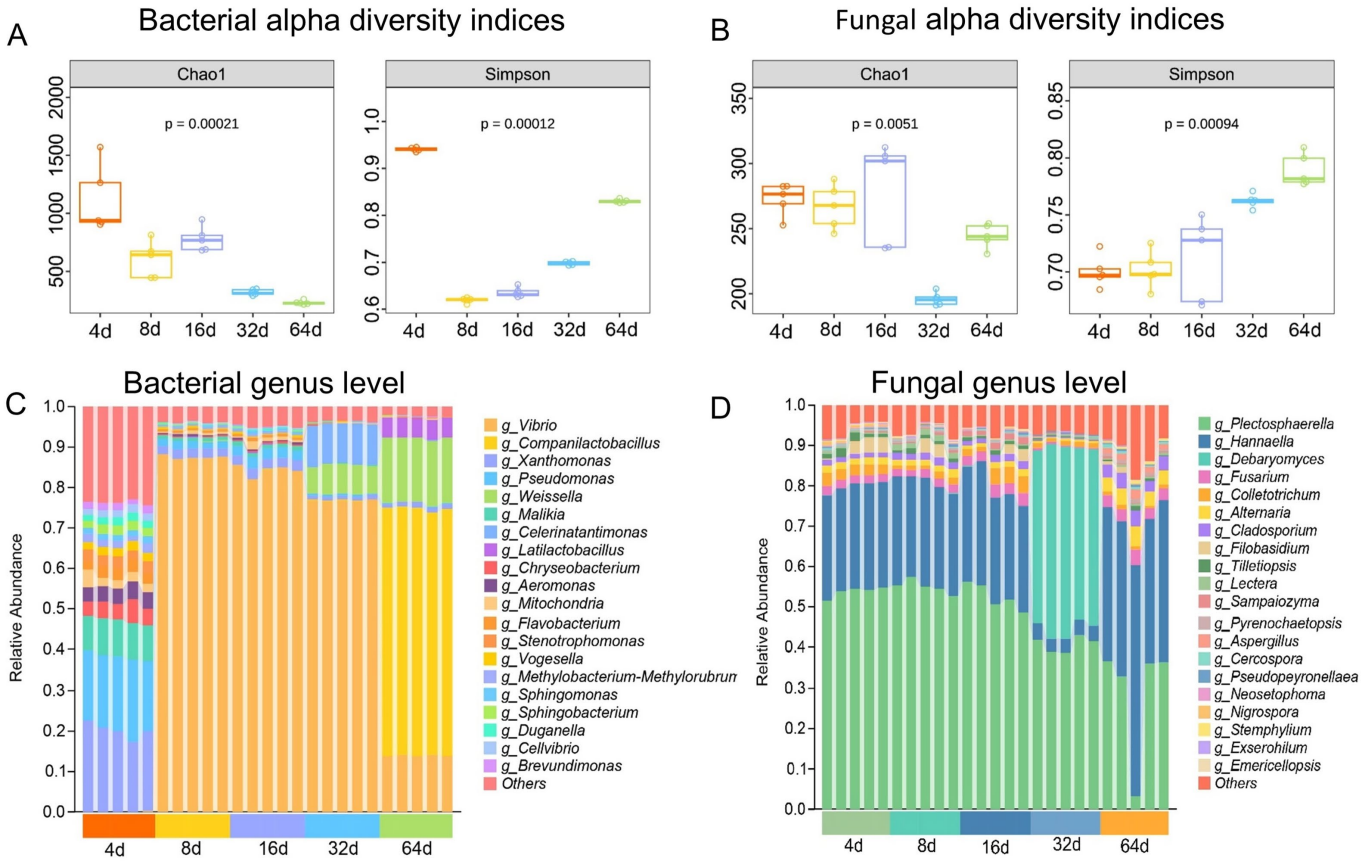
Diversity and composition of microorganisms during xuecai fermentation. **(A)** Alpha diversity of bacteria during xuecai fermentation; **(B)** Alpha diversity of fungi. **(C)** Bacterial relative abundance at genera level; **(D)** Fungal relative abundance at genera level.

The quality and unique flavor of fermented foods are related not only to the original nutrient composition but also, more importantly, to microbial metabolic activity (Song et al., 2020). To explore the link between the microbial composition and flavor substance forms of fermented xuecai in depth, microbiome sequencing technology was applied to detect changes in the microbial diversity and composition of fermented xuecai during the fermentation process. The results revealed that the bacterial Chao1 index tended to decrease during the fermentation process; the Simpson index decreased rapidly at 4 d but then increased gradually (Figure 5A), which indicated that the microbial abundance and diversity were still high at 4 d of the pickling process. However, after a longer pickling time, the bacterial abundance was suppressed, and bacterial diversity gradually converged to the flora suitable for xuecai fermentation after 8 d. Fungi also play a critical role in the fermentation of vegetables, influencing the breakdown and transformation of metabolites (Xing et al., 2024). The fungal Chao1 index did not significantly change from 4 d to 16 d but decreased significantly by 32 d. In contrast, the Simpson index tended to increase throughout the fermentation process (Figure 5B).

The UMAP analysis based on the Bray–Curtis distance showed the difference in the constitution of the microorganisms at different time points of xuecai fermentation (Supplementary Figure S3). Bacterial relative abundance dynamics at the genus and species levels in the fermented xuecai were analyzed, as shown in Figure 5C. The results revealed high relative abundances (>10%) of *Vibrio*, *Companilactobacillus*, *Pseudomonas*, *Weissella*, *Malikia*, *Celerinatantimonas*, and *Latilactobacillus*. In the early fermentation stage, *Pseudomonas* was predominant, with a relative abundance of 18.43%. The abundance of *Vibrio* was high between 16 d and 32 d, ranging from 76.60 to 86.47%, and *Companilactobacillus*, *Weissella* and *Latilactobacillus* were the main microorganisms present in the late stage of fermentation (Supplementary Table S3), with relative abundances of 61.41, 17.76, and 5.10%, respectively. At the species level, *Weissella jogaejeotgal*, *Lactobacillus ginsenosidimutans* and *Pseudomonas viridiflava* were the predominant bacteria involved in fermentation (Supplementary Figure S4; Supplementary Table S4). At the genus level, *Plectosphaerella*, *Hannaella*, *Debaryomyces*, *Fusarium*, *Colletotrichum*, and *Alternaria* were identified as the dominant fungal genera during xuecai fermentation (Figure 5D). *Debaryomyces* was more abundant at 32 d (Supplementary Table S5). *Plectosphaerella cucumerina*, *Hannaella oryzae*, and *Fusarium_equiseti* were also identified as active microorganisms at the species level (Supplementary Figure S5; Supplementary Table S6).

LEfSe was used to analyse the differentially abundant microbial taxa in xuecai at different fermentation time points. As shown in Supplementary Figure S6, the bacterial diversity and richness were highest on 4 d, which is consistent with previous results. The abundances of *Sphingobacteriaceae*, *Chromobacteriaceae*, *Pseudomonadaceae*, *Chryseobacterium*, *Acidovorax*, *Malikia*, and *Pseudomonas* differed significantly after 4 d of fermentation. The abundance of *Vibrio* showed a significant difference from that at other time points after 8 d of fermentation. The highest abundances of *Celerinatantimonadaceae*, *Celerinatantimonas*, *Halomonas*, and *Halomonas subglaciescola* were observed at 32 d of fermentation. *Lactobacillaceae*, *Latilactobacillus*, *Companilactobacillus*, *Weissella*, *Lactobacillus ginsenosidimutans*, and *Weissella jogaejeotgali* were more abundant at 64 d of fermentation (Supplementary Figure S6). Analyses of the fungi revealed significant changes in the abundances of *Hannaella*, *Colletotrichum*, *Debaryomyces*, *Fusarium* and *Paramyrothecium* (Supplementary Figure S7).

### 3.6 Correlation analysis between microorganisms and physicochemical indicators during xuecai fermentation

**Figure 6:**
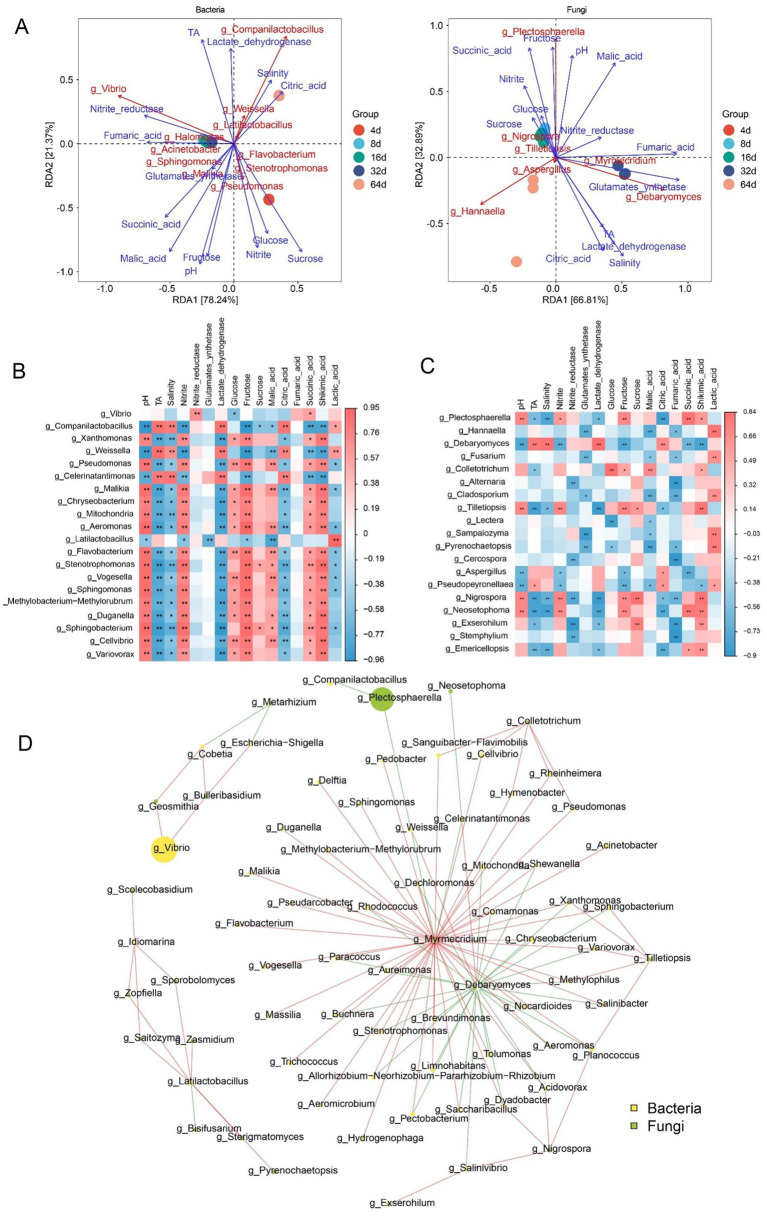
Correlations between microorganisms and physicochemical indicators during xuecai fermentation. **(A)** RDA of bacteria and fungi with physicochemical indicators. **(B)** Correlations between the bacterial community and physicochemical indicators. **(C)** Correlations between the fungal community and physicochemical indicators. **(D)** Co-occurrence network of the microbial community based on Spearman’s correlation coefficients. Red and blue lines, respectively, represent positive and negative correlations. * shows a significant association (*p* < 0.05), and ** shows a highly significant correlation (*p* < 0.01).

Physicochemical indicators during fermentation influence the dynamic changes in the microbial community (Niu et al., 2023). Therefore, we analyzed the relationships among the microbial communities and physicochemical indicators using the Mantel test. The contents of nitrite, TA, pH, lactate dehydrogenase, free sugars, organic acids (shikimic acid and citric acid) and bacteria were significantly correlated (Supplementary Figure S8). In contrast, the correlations among pH, salinity, fructose levels, organic acid levels and fungal abundance were stronger (Supplementary Figure S9). The RDA1 and RDA2 values of bacteria contributed 78.24 and 21.37%, respectively, to the total constrained variation (Figure 6A). Among the physicochemical indicators, pH, TA, and the nitrite, fructose, sucrose, malic acid, and lactic acid levels were strongly associated with bacterial communities. In particular, *Companilactobacillus*, *Weissella*, and *Latilactobacillus* had a strong positive association with TA and salinity and a strong negative association with the sugar contents, pH, organic acid contents and nitrite levels. In fungi, RDA1 and RDA2, respectively, contributed 66.81 and 32.89% to the total constrained variation (Figure 6A). Salinity and the contents of fructose, succinic acid, lactic acid and fumaric acid were strongly associated with fungal communities. In particular, *Plectosphaerella* showed a strong positive association with succinic acid levels, fructose levels, and pH. The yeasts *Debaryomyces* and *Hannaella* were significantly positively correlated with salinity and citric acid levels.

Spearman’s correlation coefficients were calculated to analyse the associations among bacteria with higher abundances and physicochemical indicators. The results revealed that, except for *Companilactobacillus*, *Weissella* and *Celerinatantimonas*, the other bacteria showed a strong positive association with pH and the nitrite, fructose and manganic acid levels (*p* < 0.05) and a strong negative association with TA and lactate dehydrogenase activity (Figure 6B). However, fungi such as *Debaryomyces* were significantly positively correlated with TA, salinity, lactate dehydrogenase activity and citric acid levels and negatively correlated with pH and the levels of nitrite, fructose, succinate and quinic acid (Figure 6C). A correlation analysis was performed at the genus level for the microorganisms in the fermented xuecai (|*R*| > 0.8, *p* < 0.05). The results of the co-occurrence network revealed that the interactions between bacteria and fungi during xuecai fermentation play a dominant role in microbial community succession (Figure 6D). *Debaryomyces* exhibited negative correlations with other microorganisms, such as *Buchnera*, *Shewanella*, *Aeromonas*, and *Methylophilus*. *Myrmecridium*, however, was positively correlated with most of the microorganisms. We conducted a Spearman association analysis on the 48 distinct flavor compounds and the dominant components of the microbiota (|*r*| > 0.6, *p* < 0.05) (Supplementary Figure S10). The results revealed that *Companilactobacillus*, *Weissella* and *Debaryomyces* exhibited strong positive correlations with the levels of amino acids, citric acid, N-acetyl-homoserine lactone, methyl-trans-cinnamate, acetol, mannitol, 2-nonenal, dihydroxyacetone and syringaldehyde (*p* < 0.05).

### 3.7 Association network analysis between the microbial community and flavor compounds

**Figure 7:**
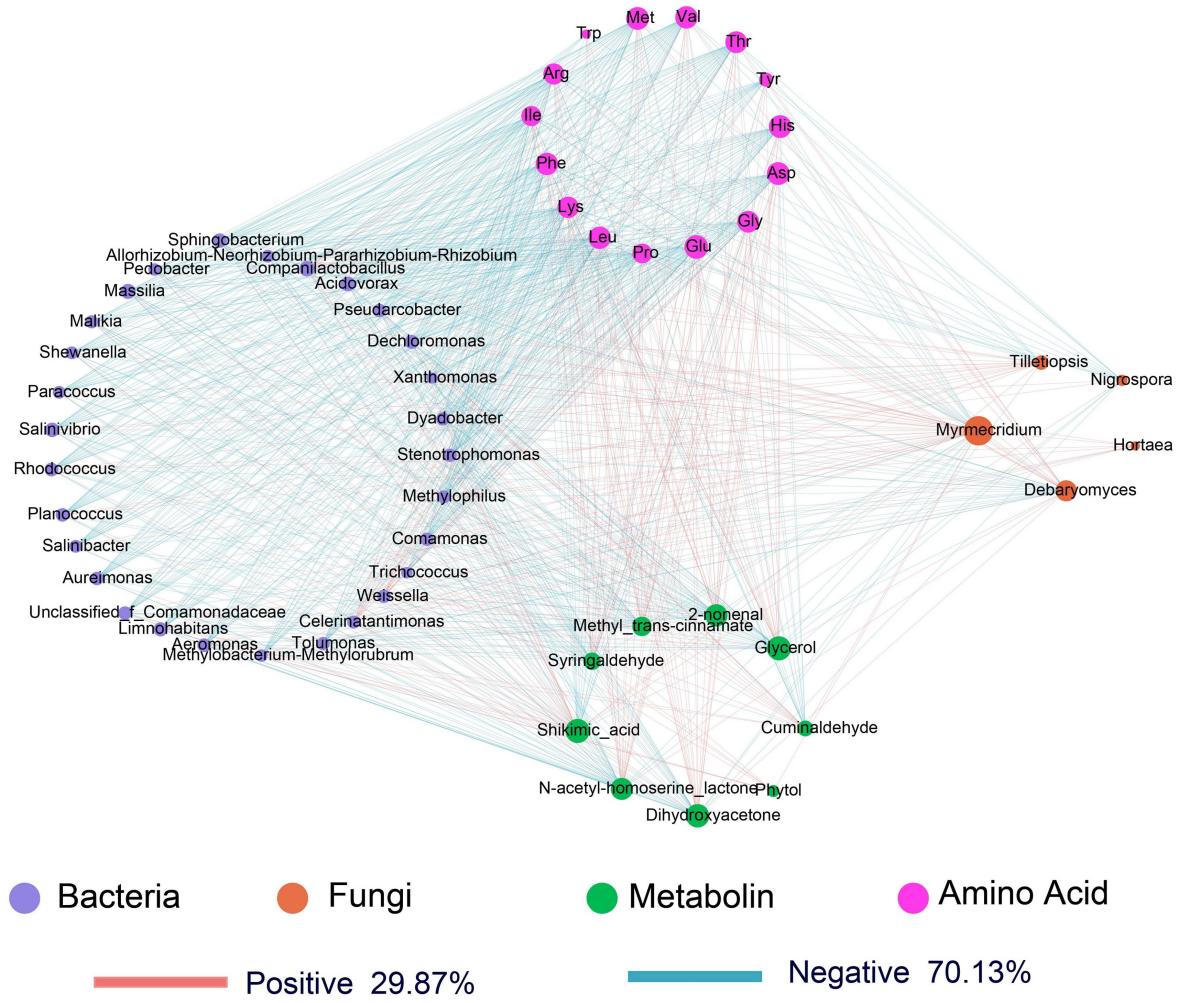
Microbial metabolic network showing correlations between the microbiota and core metabolites during xuecai fermentation. The upper circles represent major amino acids, and the lower circles represent major volatile flavor compounds of fermented xuecai. The left circle denotes major bacteria, and the right circle denotes major fungi. The red line denotes a positive correlation coefficient, while the blue line denotes a negative correlation coefficient.

An association analysis (|*r*| > 0.8. *p* < 0.05) of key microorganisms (5 fungi and 29 bacteria) with flavor compounds in fermented xuecai indicated that bacteria had a greater effect on flavor compound formation than fungi (Figure 7); the correlation data tables are shown in Supplementary Tables S7, S8. Overall, most bacteria showed a negative correlation with the contents of amino acids and organic flavor compounds, while displaying a positive correlation with the contents of shikimic acid and cuminaldehyde. However, *Companilactobacillus* and *Weissella* were positively correlated with the contents of amino acids, N-acetyl-homoserine lactone, 2-nonenal, glycerol, syringaldehyde, methyl-trans-cinnamate and dihydroxyacetone but negatively correlated with the cuminaldehyde content. With respect to fungi, *Debaryomyces* was negatively correlated with shikimic acid levels but positively correlated with the contents of amino acids, glycerol, N-acetyl-homoserine lactone, and dihydroxyacetone. Therefore, flavor-related amino acids and flavor substances such as aldehydes, esters, and ketones were strongly correlated with the abundance of microorganisms, especially *Companilactobacillus*, *Weissella*, and *Debaryomyces*.

**Figure 8:**
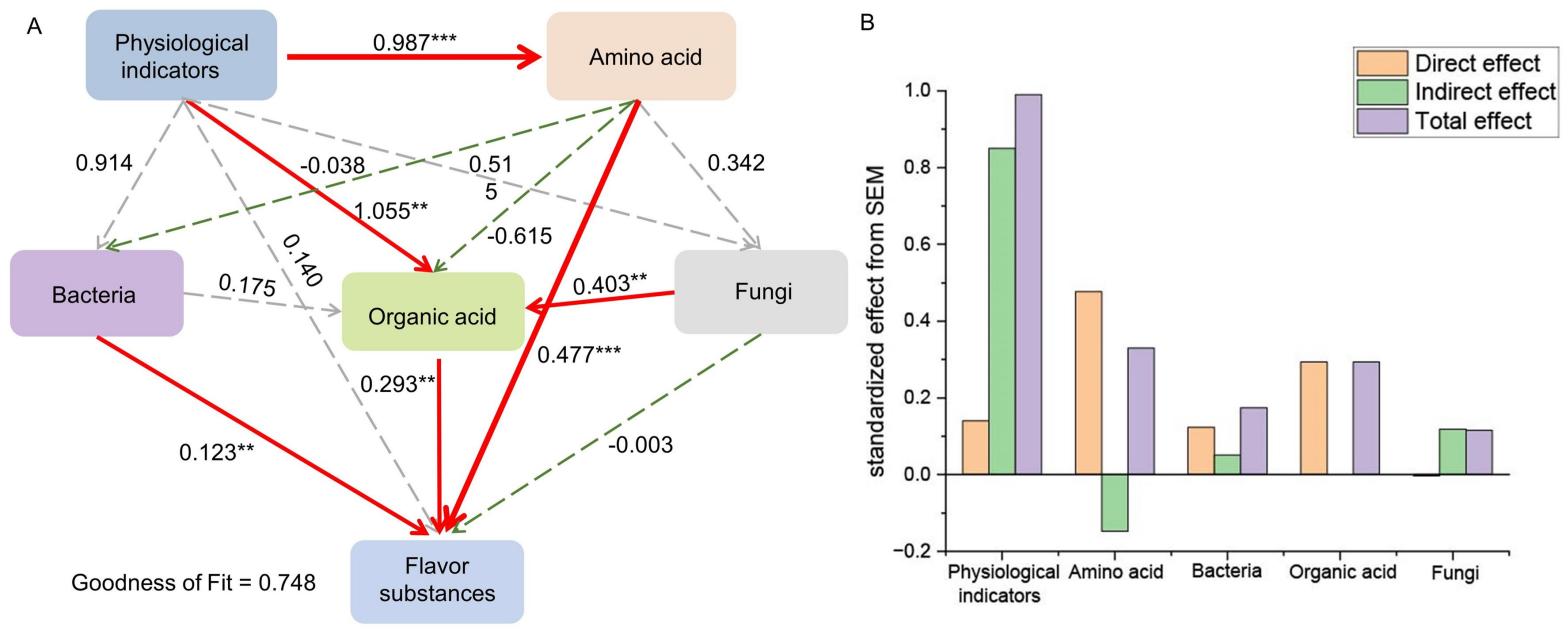
**(A)** Structural equation modeling (SEM) between physiological indicators, microbial communities, and key flavor substances; numbers next to the arrows are correlation coefficients. * shows *p* < 0.05, ** shows *p* < 0.01, and *** shows *p* < 0.001; the more asterisks there are, the stronger the association. **(B)** Direct and indirect effects of physiological indicators and microorganisms on key flavor substances.

In addition, the formation of flavor substances in fermented vegetables is related to numerous factors, including physicochemical indicators, acidity values and microbial communities (Li et al., 2025; Martinez-Villaluenga et al., 2009). The structural equation modeling (SEM) results indicated that physicochemical indicators exhibited a significant positive correlation with the contents of amino acids and organic acids. Furthermore, bacteria, amino acids, and organic acids made a significant contribution to the production of flavor compounds (Figure 8A). Physicochemical indicators contributed to the production of flavor compounds mainly indirectly by affecting changes in the levels of organic acids and amino acids, whereas bacteria contributed directly to the formation of flavor substances (Figure 8B).

## 4 Discussion

Microbial fermentation drives the biosynthesis of a diverse array of flavor compounds in xuecai, which collectively contribute to its distinctive flavor and taste. Thus, elucidating the intricate correlations between the microbial community and flavor-active metabolites is of paramount importance. Generally, microorganisms proliferate vigorously during the early stage of vegetable fermentation, causing dynamic changes in both the microbial community and physicochemical properties. As the fermentation progresses into the late stage, the microbial community stabilize (Zabat et al., 2018). Therefore, this study analyzed the physicochemical properties, metabolites, and microbial community dynamics of xuecai fermentation on days 4, 8, 16, 32, and 64. Meanwhile, establishing to elucidate the mechanisms driving flavor compound formation. This study provides theoretical support for optimizing and enhancing fermentation quality.

The distinctive flavor profiles developed during vegetable fermentation are predominantly determined by the microbial communities involved in the fermentation process, the composition of which is readily modulated by a variety of environmental factors. Previous studies have found that Laotan Suancai and black rice wine had not yet produced salt and acid by the 0th day of fermentation, while the levels of flavor amino acids and organic acids were also extremely low. However, under the action of microorganisms during fermentation, the physicochemical indicators underwent dynamic changes (Tang and Peng, 2024; Xiong et al., 2024). Our study found that during the fermentation of xuecai from day 4 to 64, the TA increased gradually, while the pH decreased correspondingly. This initial increase is attributed to the fact that acid-producing bacteria such as lactic acid bacteria convert sugar into organic acids such as acetic acid and lactic acid during the vegetable fermentation process, resulting in an increased TA (Tian et al., 2021). The salinity gradually increased, whereas the nitrite content first increased but then decreased. Salt is an important component in the fermentation of xuecai, and a high concentration of salt not only inhibits the propagation of harmful microorganisms but also enhances the flavor of pickles (Tang et al., 2024). The dehydration of vegetables due to water osmosis during fermentation leads to salt accumulation. The proliferation of acid-producing bacteria such as lactobacilli led to the accumulation of organic acids, which neutralized the nitrite in the pickled vegetables (Oh et al., 2004). Nitrite reductase (NiR) serves as the pivotal metabolic enzyme in the decomposition of nitrite during the vegetable fermentation process (Xu et al., 2024). The electronic nose analysis revealed that the contents of sulfur-containing compounds gradually increased during the late stage of fermentation. Cruciferous vegetables, such as *Brassica oleracea*, are inherently rich in sulfur-containing precursors such as glucosinolates and cysteine. During fermentation, the degradation of these precursors is influenced by LAB, generating volatile sulfur compounds (e.g., dimethyl sulfide, allyl isothiocyanate, and thiols), which become a significant source of the distinctive flavor of fermented vegetables (Friedrich et al., 2022). The electronic tongue analysis indicated a significant increase in sourness during fermentation. This increase in sourness can be attributed to the generation of various flavor compounds, such as amino acids, during vegetable fermentation (Sun et al., 2023). In addition, LAB metabolized sugars into pyruvate, which was subsequently converted into lactic acid, leading to a significant increase in acidity (Alan, 2019).

The unique flavor and texture of fermented xuecai are derived from the flavor substances generated during the fermentation process. In this study, we detected a rich variety of both volatile and nonvolatile flavor compounds through metabolomics. Amino acids, important taste-active compounds and aroma precursors, reached their maximum concentration on the 32 d of fermentation. The changes in amino acid contents were attributed mainly to the metabolic activities of microorganisms, such as LAB and yeast (Weiller and Radler, 1976). Protein hydrolases secreted by microorganisms can hydrolyse the proteins in vegetables into peptides and free amino acids, which leads to an increase in the FAA content during fermentation. Previous studies have reported that red light can also promote the accumulation of lysine and proteins (Wang J. et al., 2024). In addition, these amino acids can be further converted into aromatic compounds (e.g., sulfur-containing compounds, aldehydes, and alcohols) through transamination and elimination reactions (Jung et al., 2014; Smit et al., 2005). The amino acid content reached a maximum value on the 32 d of xuecai fermentation but then decreased thereafter. This result may be related to the prolonged fermentation time for microbial uptake and utilization. For example, the prolonged fermentation of fermented cucumber triggers spoilage and affects its quality (Franco and Pérez-Díaz, 2012). Therefore, 32 d of fermentation is the most suitable duration for maintaining the flavor-related amino acids of fermented vegetables. In addition to amino acids, organic acids are key nonvolatile components produced during xuecai fermentation. Previous studies have shown that malic acid and fumaric acid are important metabolites in fermented vegetables and play crucial roles in maintaining their flavor profile (Hou et al., 2023). Citric acid, succinic acid, and shikimic acid serve as intermediates in the microbial glycolytic, tricarboxylic acid (TCA), and shikimate pathways for the synthesis of flavor-related amino acids (Peng et al., 2025b). In addition, organic acids have been found to be useful for degrading nitrite during the fermentation of sauerkraut in Northeast China (Xu et al., 2024). Thus, the fermentation broth is enriched with a wide range of organic acids, which are not only converted into flavor-related amino acids but also degrade nitrites.

Volatile organic compounds serve as the primary source of flavor during xuecai fermentation. We obtained 46 different metabolites via nontargeted metabolomics. Nontargeted metabolomics was also used to detect phenolic compounds in fermented mung beans. It was found that solid-state fermentation significantly enhanced the synthesis of phenolics, and the metabolic mechanism underlying phenolic compounds was further elucidated (Lang et al., 2025). Our research revealed that alcohols and esters were the predominant volatile compounds, similar to the metabolites produced during the fermentation of black rice wine (Tang and Peng, 2024). Among these metabolites, the contents of glyceraldehyde, fructose 2,6-biphosphate, 3,4-methylenedioxycinnamaldehyde, iberin, butyrolactone, 2(3H)-furanone, and other compounds gradually decreased during fermentation. The contents of most alcohols and esters, such as tyrosol, N-acetyl-homoserine lactone (AHL) and 3-methyl-3-buten-1-ol, gradually increased during the later stages of fermentation. Iberin belongs to the class of isothiocyanate compounds and contributes to the pungent odor of mustard (Zhao et al., 2007; Zhao et al., 2022). In contrast, the microorganisms involved in fermentation gradually decompose iberin, which is similar to previous findings in xuecai (Liu et al., 2021). Some microorganisms can produce acetol via the methylglyoxal pathway (Zhu et al., 2013), which can be further converted to lactic acid or acetolactic acid (Xiao et al., 2022). By modulating microbial metabolic activities, AHLs indirectly influence the formation of organic acids and ester flavor compounds (de Almeida et al., 2017). Tyrosol and resveratrol are phenolic compounds. Previous studies have identified tyrosol as a metabolite of tyrosine produced during wine fermentation (Boronat et al., 2018). Homologs of 3-methyl-3-buten-1-ol have been identified as key compounds responsible for the malty aroma of cheese, contributing to its pleasant flavor profile (Meng et al., 2021). These results demonstrate that volatile compounds are synthesized by microorganisms through various metabolic activities to form flavor substances, among which tyrosol, syringaldehyde and 3-methyl-3-buten-1-ol contribute significantly to the generation of flavor substances in fermented xuecai.

Based on amplicon sequencing technology, we investigated the dynamic changes in the microbial community during xuecai fermentation. Bacterial diversity gradually converged to the flora suitable for xuecai fermentation after 8 d, and upon the completion of fermentation, the fungal abundance decreased while the diversity increased, which is consistent with the results of research on fermented radishes and Bulang pickled tea (Yang et al., 2022; Zhou et al., 2024). An analysis of the relative abundance of microorganisms at the genus level revealed that *Vibrio*, *Companilactobacillus*, *Pseudomonas*, *Weissella*, *Malikia*, *Celerinatantimonas*, *Latilactobacillus*, *Plectosphaerella*, *Hannaella*, *Debaryomyces*, *Fusarium*, *Colletotrichum*, and *Alternaria* were the dominant genera. *Companilactobacillus*, *Weissella*, *Latilactobacillus*, and *Debaryomyces* were the dominant microorganisms during the late stage of fermentation. Specifically, *Companilactobacillus* can enhance food safety and flavor by producing organic acids and antimicrobial substances (Yang et al., 2025). *Companilactobacillus* isolated from Thai fermented fish has been identified as a potential probiotic with cholesterol-lowering and immunomodulatory activities (Kingkaew et al., 2023). It relies mainly on the LuxS/AI-2 quorum sensing system for cell–cell communication, thereby promoting biofilm maturation and enhancing tolerance to acid, bile salts, and osmotic stress (Papadimitriou et al., 2016). Biofilm formation is synergistically regulated by multiple genes, and ARTP mutagenesis can efficiently induce gene mutations in the spoilage bacterium *Pseudomonas fluorescens* PF08, serving as an effective tool for screening biofilm-related functional mutants and mining biofilm regulatory genes (Wang et al., 2025). *Weissella* is also a genus of LAB. Multiple genes associated with probiotic functions have been identified in *Weissella*, and these genes contribute to the maintenance of human intestinal health (Rocha et al., 2025). Furthermore, *Debaryomyces* has been detected in cheese and fermented soy sauce, which produces a variety of volatile compounds that contribute to the flavor of fermented products (Gori et al., 2012; Jeong et al., 2022). The quorum sensing function of *Debaryomyces* is mediated mainly by aromatic alcohols such as phenylethanol and tyrosol, which regulate cell adhesion and biofilm formation (Gori et al., 2011). Therefore, these key microorganisms may also be critical for flavor production in pickled xuecai. Notably, *Halomonas*, a halophilic genus, was highly abundant on day 32 of fermentation, consistent with the maximum salt concentration observed at this stage and is consistent with previous findings in *Brassica juncea* fermentation (Liu et al., 2021). LEfSe showed that the distribution of signature bacteria was heterogeneous across different fermentation time points, which may be closely associated with fermentation time and environmental conditions. In summary, *Weissella*, *Companilactobacillus*, *Latilactobacillus*, *Debaryomyces*, and *Hannaella*, which constitute the dominant microbiota in the fermentation process of xuecai, may be the key microorganisms involved in the formation of quality and flavor substances in fermented xuecai.

Dynamic changes in the microbial community are affected not only by environmental factors but also by interactions between microorganisms, which play a key role in influencing its stability. Furthermore, the correlation analysis between microorganisms and environmental factors indicated that TA, salinity, nitrite levels and organic acid contents were the main driving factors of bacterial community succession during xuecai fermentation and strongly influenced the changes in the LAB community, especially *Companilactobacillus*, *Weissella* and *Latilactobacillus.* Previous studies have reported that organic acids modulate the spatiotemporal distribution of microbial genera such as *Weissella* and *Bacillus* through pH regulation and the induction of enzyme activity, thereby driving the microecological differentiation of high-temperature Daqu (Yang et al., 2024). Nitrite can suppress the growth of *Enterococcus* and regulate the abundance of lactic acid bacteria (LAB) while simultaneously reducing the biogenic amine content (Wu et al., 2024). With respect to fungi, physicochemical indicators such as salinity and the contents of fructose, succinic acid and lactic acid were strongly correlated mainly with the abundances of *Plectosphaerella*, *Debaryomyces* and *Hannaella*. Notably, the microbial co-occurrence network revealed that the abundance of *Debaryomyces* exhibited negative correlations with other microorganisms, and this negative correlation is likely attributable to competitive interactions among these microorganisms for nutrient resources (Becker et al., 2012). However, the abundance of *Myrmecridium* was positively correlated with the abundances of most of the microorganisms, suggesting that it works synergistically with other microorganisms to maintain the stability of the microbial community of fermented xuecai. These microorganisms play a crucial role in the formation of flavor during food fermentation. Spearman correlation heatmap analysis showed that *Companilactobacillus*, *Weissella* and *Debaryomyces* were positively associated with the contents of amino acids, citric acid, N-acetyl-homoserine lactone, methyl-trans-cinnamate, acetol, mannitol, 2-nonenal, dihydroxyacetone and syringaldehyde. However, the observed associations between microorganisms and metabolites may arise from indirect effects. Environmental factors such as nutrients, moisture, and pH can also influence the microbiota–metabolite network by regulating key metabolites (Baker et al., 2024; Ji et al., 2025). Previous studies have also shown that *Companilactobacillus* and *Weissella* contribute to the formation of flavor substances in fermented kimchi (Xiao et al., 2018). *Debaryomyces* were identified as active microorganisms during the early and late fermentation stages of xuecai, producing esters and alcohols, respectively, and contributing to the formation of flavor substances in fermented foods (Güneşer et al., 2015; Wu et al., 2022). Methyl-trans-cinnamate has a cinnamon-like aroma, which can enhance the flavor of fermented vegetables. Furthermore, it has antimicrobial activity, helping to inhibit the growth of spoilage microbes and thereby prolonging the storage life of fermented products (Huang et al., 2009). 2-Nonenal enhances the aroma of fermented suansun and plays a crucial role in the formation of its unique flavor (Li et al., 2025). In addition, 2-nonenal contributes to a fatty aroma and is recognized as a key aroma compound in wheat bread (Vermeulen et al., 2007). These findings provide further evidence that *Companilactobacillus*, *Weissella*, and *Debaryomyces* are key microorganisms involved in the fermentation of xuecai and may promote the formation of flavor substances. Members of the genus *Companilactobacillus* can metabolize citrate and malate. Specifically, *Companilactobacillus crustorum LMG 23699* can generate buttery aroma compounds, including diacetyl, via citrate catabolism (Comasio et al., 2021). It is known that *Weissella confusa* and *Weissella cibaria* can produce high levels of oligosaccharides and exopolysaccharides, which are mainly used in the production of bread and functional cereal-based fermented beverages (Fusco et al., 2015). Studies have shown that the use of a mixed starter culture composed of *Companilactobacillus* and *Wickerhamomyces* can guide sourdough production, yielding sourdough that is rich in esters and characterized by fruity notes (Pradal et al., 2025). The findings of this study provide a theoretical basis and technical support for the standardization and industrial production of fermented products. In future work, the individual or mixed strains identified in this study could be developed as starter cultures for industrial application to further improve fermentation quality.

In addition to these key microorganisms, nutritional components and fermentation conditions also directly or indirectly modulate the generation of flavor substances and the quality of fermented xuecai. The initial physicochemical indicators of fermentation provide a specific environment for microorganisms to live in, which is the basis for the production of fermented flavor substances, and the metabolic activities of microorganisms can lead to changes in the fermentation environment. For example, the quality of fermented vegetables can be influenced by changes in microorganisms and physicochemical indicators during the storage of fermented cucumbers (Franco and Pérez-Díaz, 2013; Sawada et al., 2021). These results provide valuable insights for strain selection in industrial production and for improving the flavor and quality of fermented xuecai. Future studies could establish pure cultures of these strains and analyse their metabolic products to clarify their direct contributions to flavor formation.

## 5 Conclusion

In this research, the physicochemical indicators, flavor metabolites and microbial communities of xuecai during fermentation were systematically investigated by combining untargeted metabolomics and microbiomics. The results indicated that the contents of sulfur compounds in xuecai gradually increased during fermentation, the acidity increased, and the nitrite content gradually decreased. Flavor-related amino acids, such as bitter and sweet amino acids, were most abundant at 32 d. We detected 113 volatile flavor compounds using GC–TOF–MS, among which aldehydes and alcohols, including syringaldehyde, 2-nonenal, tyrosol, 3-methyl-3-buten-1-ol, acetol, and resveratrol, contributed spicy, plant-like, and fruity aromas and played important roles in the development of the characteristic flavor of fermented xuecai. The microbiome analysis revealed that 13 microbial genera were key contributors to fermentation. A correlation analysis of the relationships among microbes and flavor compounds further revealed that *Companilactobacillus*, *Weissella*, *Latilactobacillus*, *Debaryomyces*, and *Hannaella* are crucial for the formation of flavor compounds in xuecai. Our study provides comprehensive insights into microbial community succession and flavor dynamics in xuecai fermentation, as well as significant implications for improving the quality of fermented food and facilitating its industrial production.

## Data Availability

Data will be made available on request. The raw sequencing data (16S rRNA gene amplicon) were deposited to NCBI Sequence Read Archive (SRA) in BioProject under accession numbers PRJNA1332375. The metabolomics data have been deposited to MetaboLights repository under accession numbers MTBLS13307.

## Footnotes

1
https://www.genescloud.cn
