# Supplementary material for: Multi-omics analysis reveals the mechanism underlying microbial-driven flavor formation in fermented xuecai (Brassica juncea var. multiceps)
Source: Front Microbiol. 2026 Mar 23;17:1784956. doi: 10.3389/fmicb.2026.1784956 (PMC13050910; doi:10.3389/fmicb.2026.1784956)
Supplement: Supplementary file 1 [file Data_Sheet_1.docx]

**Multi-omics analysis reveals the mechanism underlying microbial-driven flavour formation in fermented xuecai (*Brassica juncea* var. *multiceps*)**


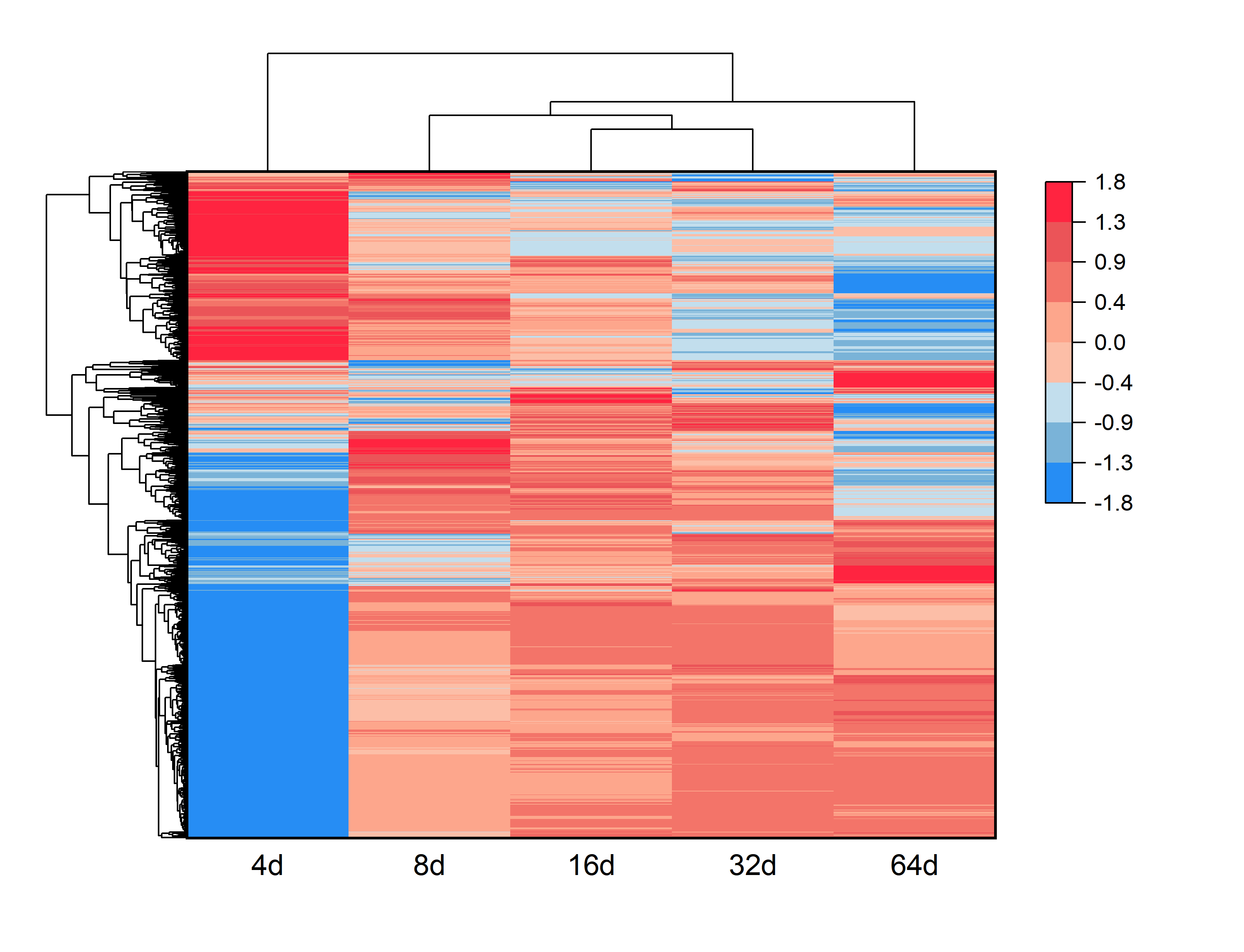


**Supplementary Figure S1** Hierarchical clustering heat map of all non-volatile metabolites in fermented xuecai


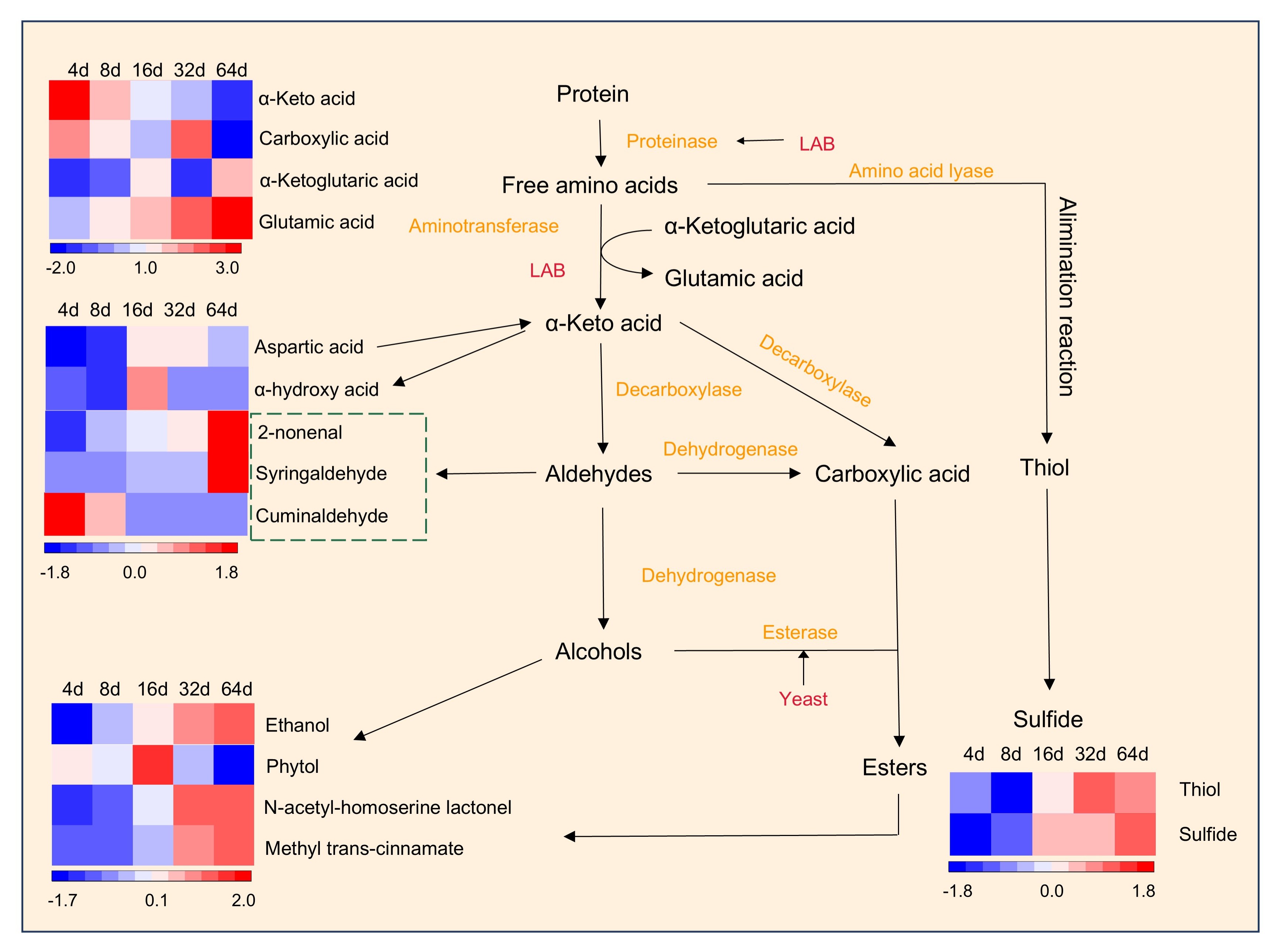


**Supplementary Figure S2** Analysis of flavour compounds metabolic pathway during xuecai fermentation


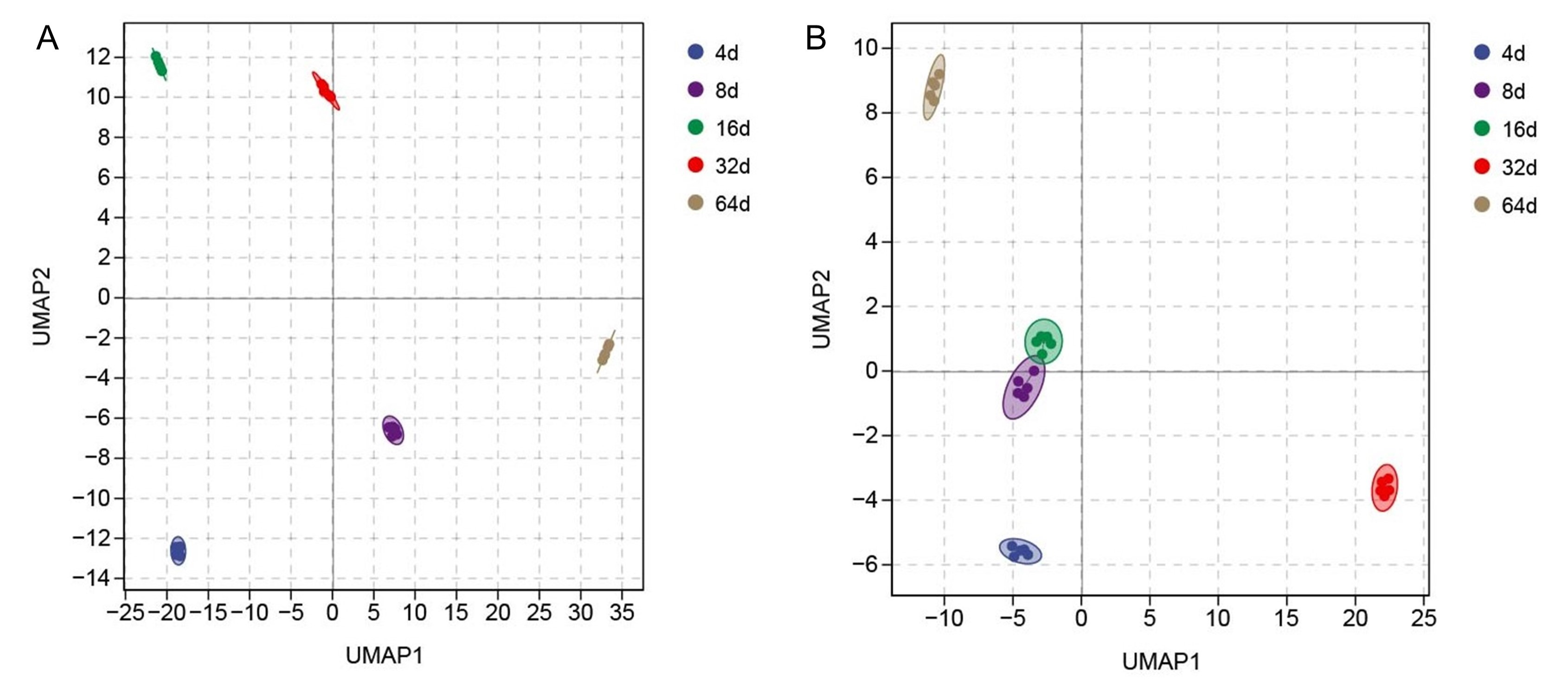


**Supplementary Figure S3** (**A**) UMAP plot of bacterial community of fermented xuecai; (**B**) UMAP plot of the fungal community of fermented xuecai.


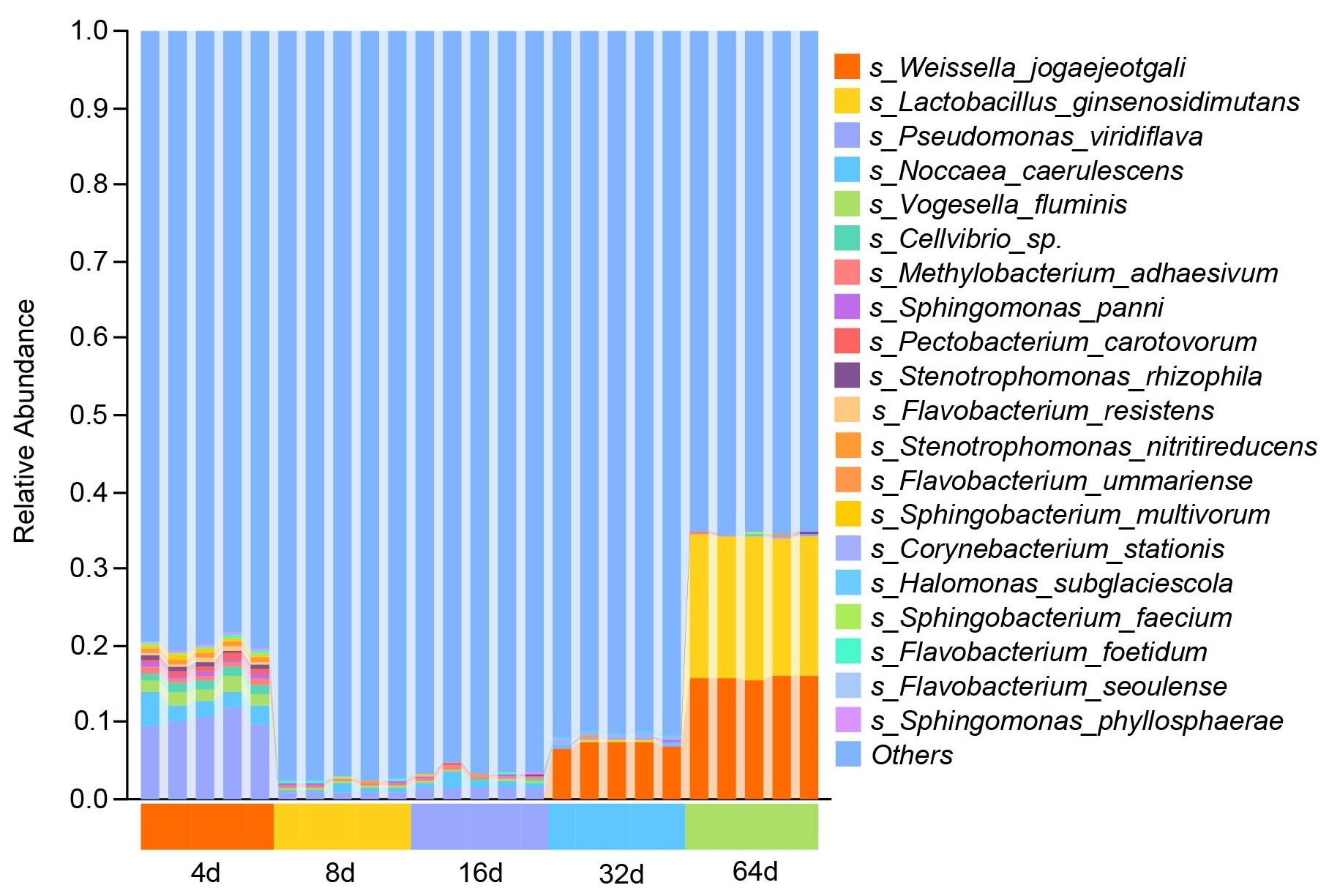


**Supplementary Figure S4** Relative abundance of bacteria at the species level


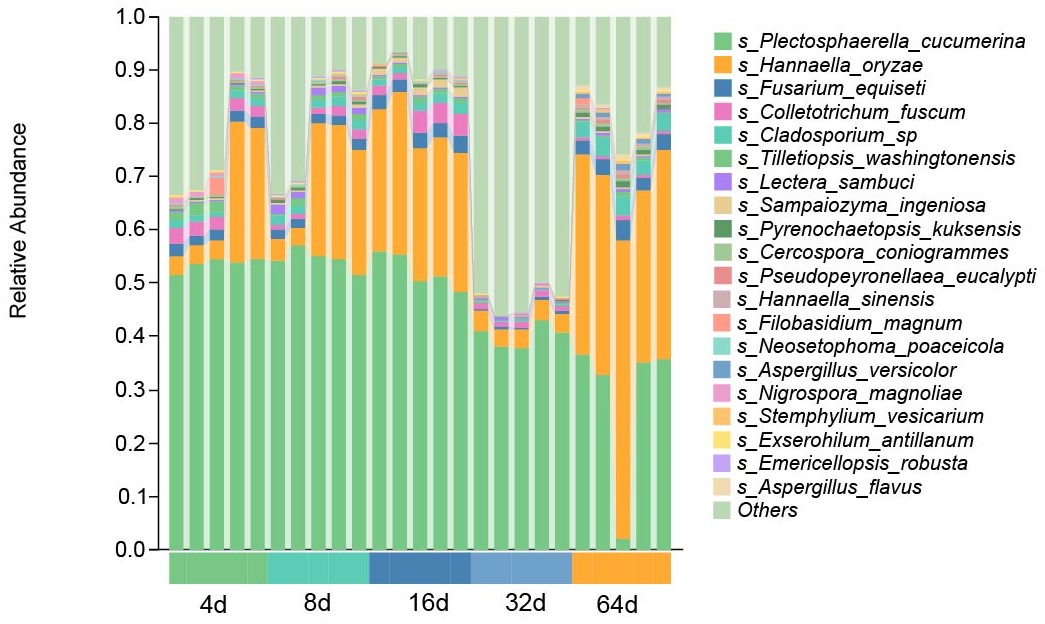


**Supplementary Figure S5** Relative abundance of fungi at the species level


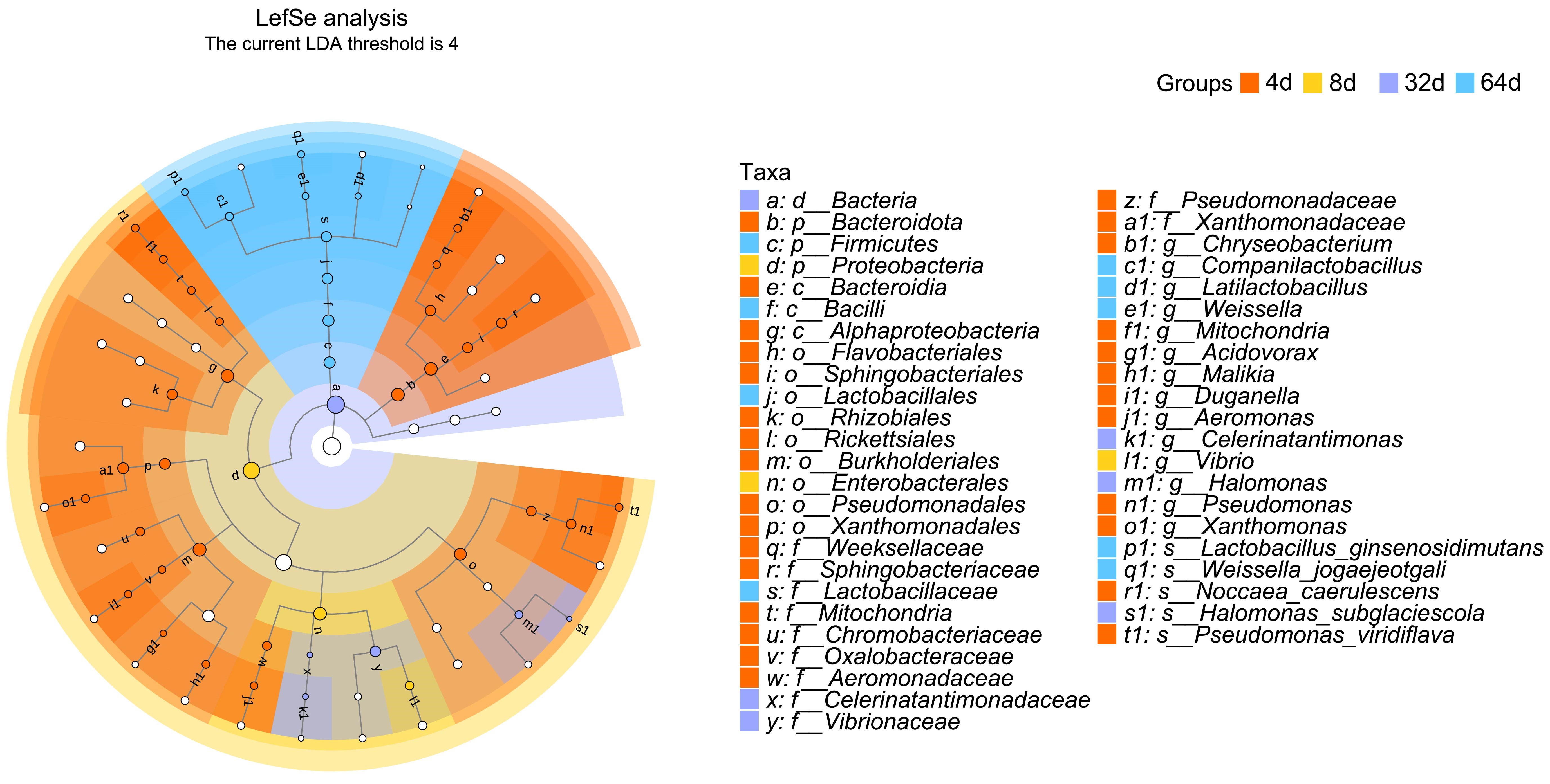


**Supplementary Figure S6** The signature functions of bacterial communities at each fermentation stage based on LEfSe analysis.


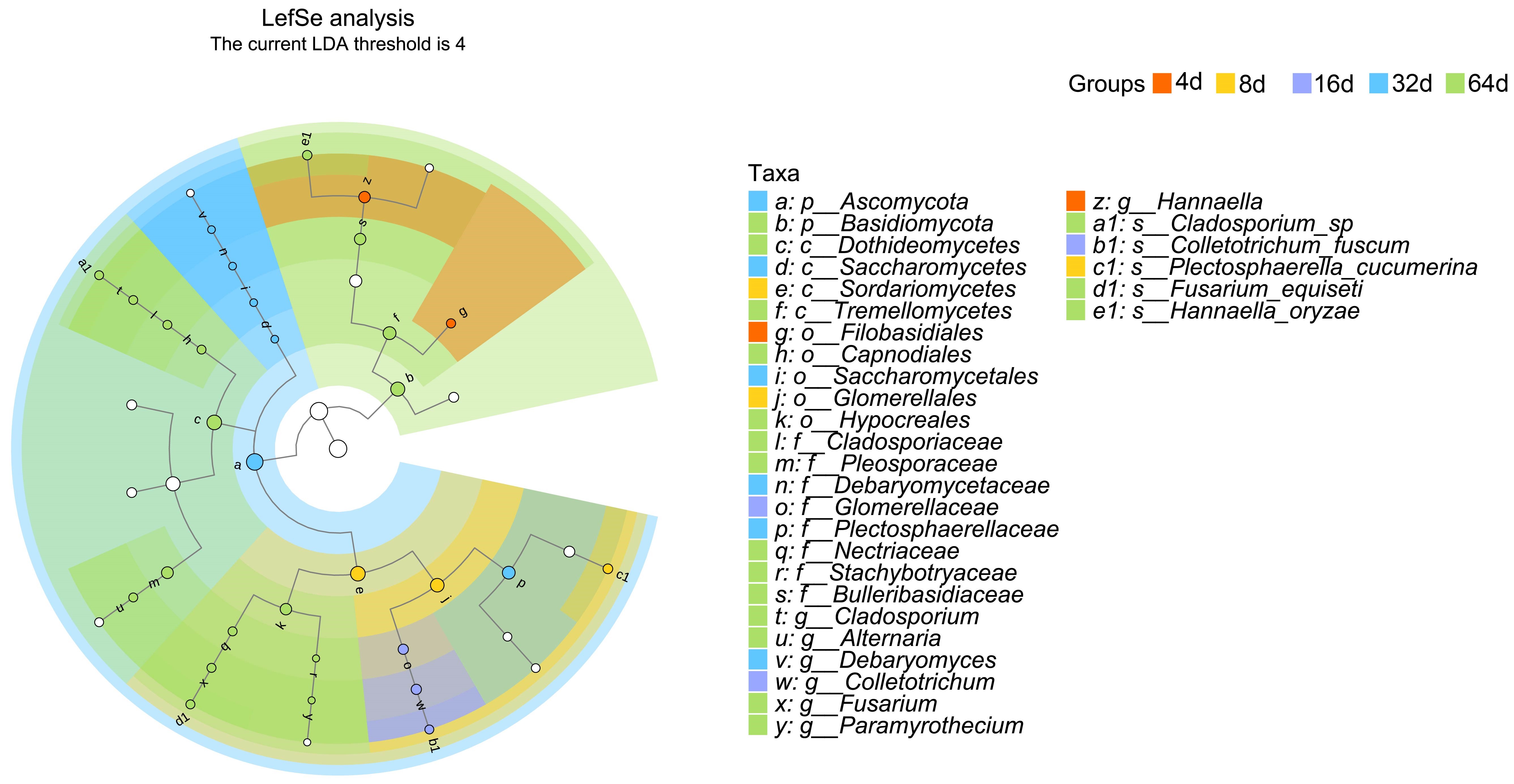


**Supplementary Figure S7** The signature functions of fungal communities at each fermentation stage based on LEfSe analysis.


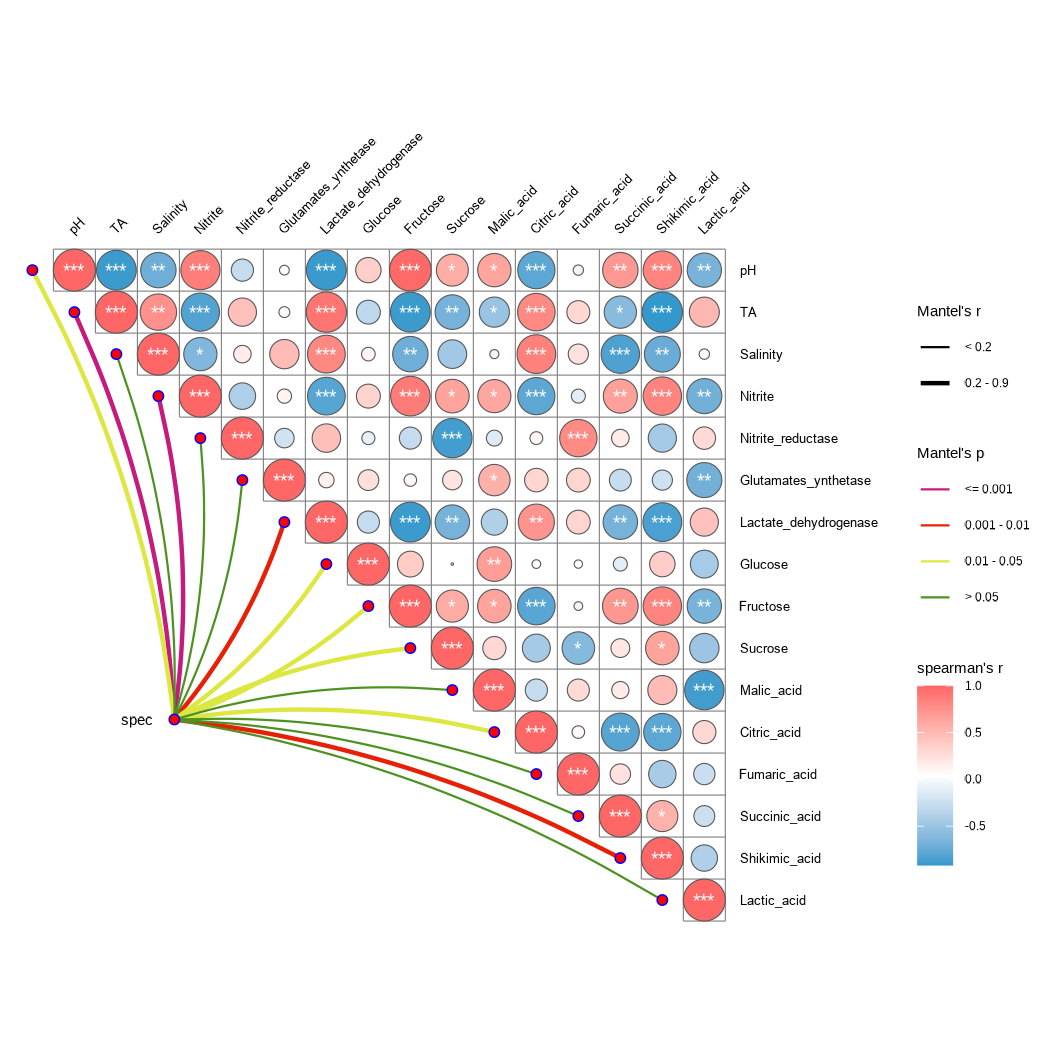


**Supplementary Figure S8** Analysis of bacterial associations with physicochemical indicators by mantel test. *p < 0.05，**p < 0.01，***p < 0.001.


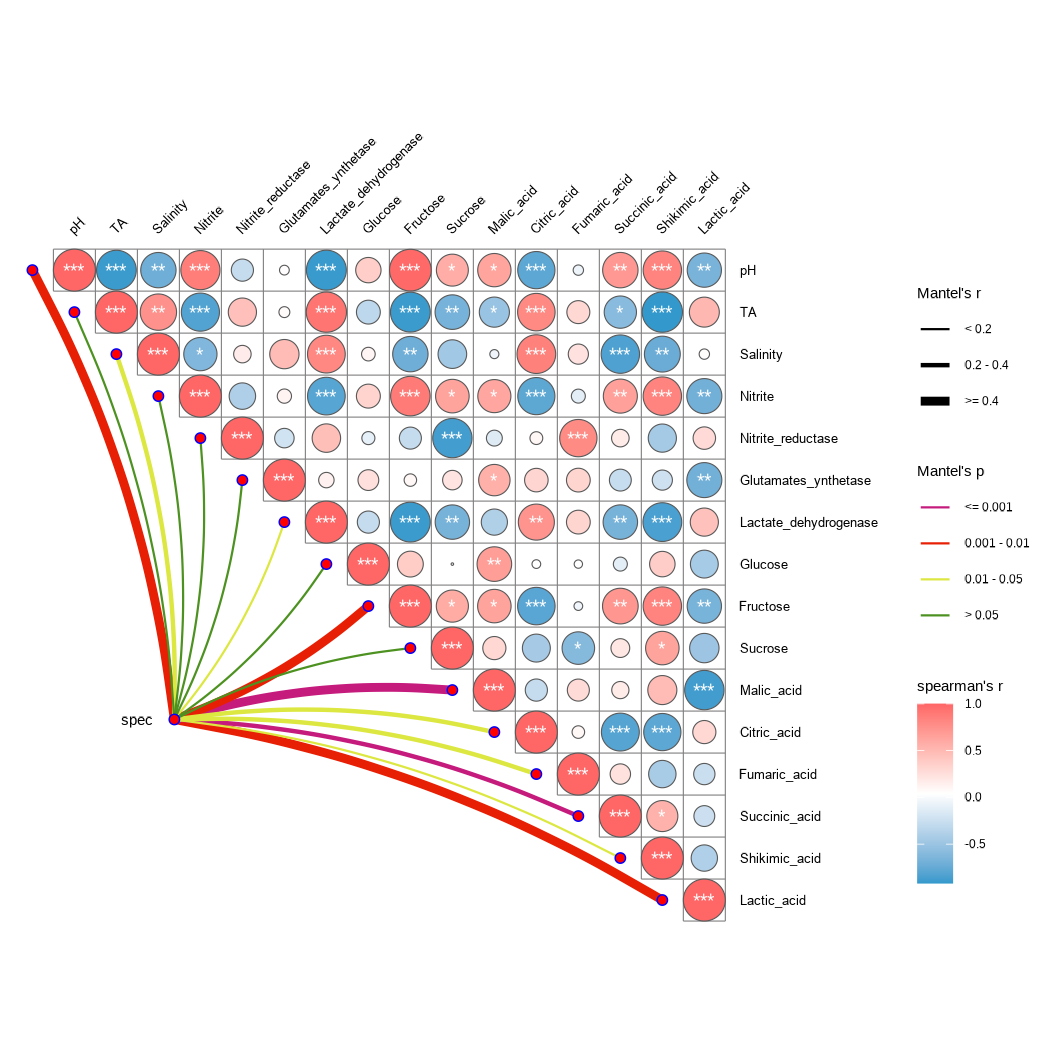


**Supplementary Figure S9** Analysis of fungal associations with physicochemical indicators by mantel test. *p < 0.05，**p < 0.01，***p < 0.001.


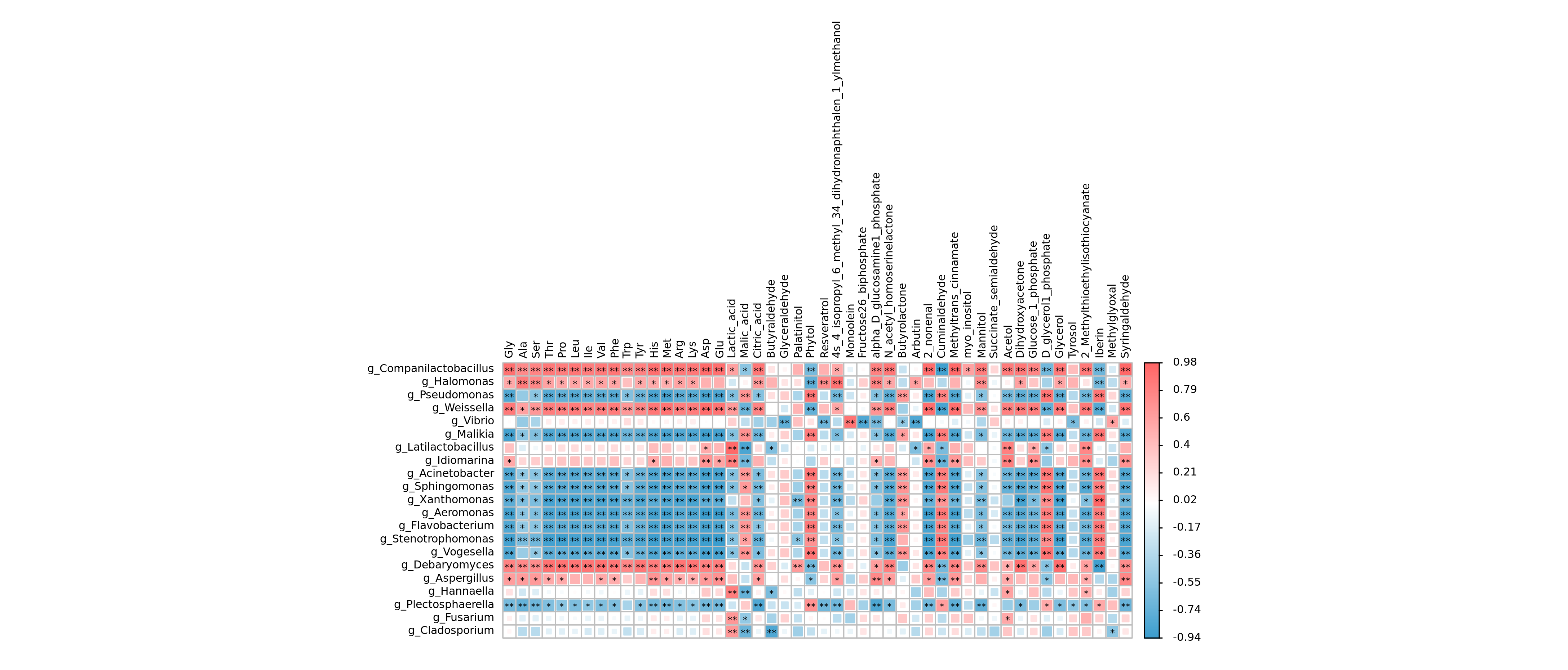


**Supplementary Figure S10** Correlation between differential characteristic flavor compounds and dominant microbiota based on Spearman correlation efficient (|r| > 0.8, p < 0.05).
